# Chronic wound management: a liquid diode-based smart bandage with ultrasensitive pH sensing ability

**DOI:** 10.1038/s41378-024-00801-6

**Published:** 2024-12-16

**Authors:** Xueqi Wang, Jing Cheng, Han Wang

**Affiliations:** 1 School of Biomedical Engineering, Tsinghua University, Beijing, 100084 China; 2 National Engineering Research Center for Beijing Biochip Technology, Beijing, 102206 China

**Keywords:** Biosensors, Biosensors

## Abstract

Chronic wounds, which require prolonged healing periods, pose significant impacts on individuals with diabetes, vascular diseases, and high blood pressure. Simultaneous drainage and monitoring of wound exudate are vital for advanced wound management. However, recently reported smart dressings either lack integration of wound cleaning and monitoring functions or fail to achieve dynamic in situ monitoring of wound status, which hinders their ability to meet the demands of wound care. In this study, a smart bandage is introduced, which integrates a biocompatible liquid diode membrane with an ultrasensitive 3D polyaniline mesh (M-PANI)-based pH biosensor. The smart bandage allows for unidirectional drainage of wound exudate while dynamically sensing the wound pH environment. Specifically, the proposed smart bandage effectively cleans excessive wound exudate while providing real-time information on the wound status during the drainage process. The M-PANI-based pH biosensor demonstrates a high sensitivity of 61.5 mV/pH and a wide pH detection range from 4.0 to 10.0, encompassing the pH range of normal and infected wounds. Moreover, the sensing module exhibits excellent stability after 48 hours of dynamic testing and 28 days of storage, with only a 4.8% decline in the detected signal, and high repeatability with a device-to-device relative standard deviation (RSD) of 3.1%. To evaluate the practicality of this smart bandage, simulated skin and rats have been employed, and the results indicate the immense potential of this smart bandage for clinical applications. In conclusion, the present smart bandage demonstrates considerable promise for wound exudate cleaning and monitoring in advanced wound care and offers a promising method for home-based wound management.

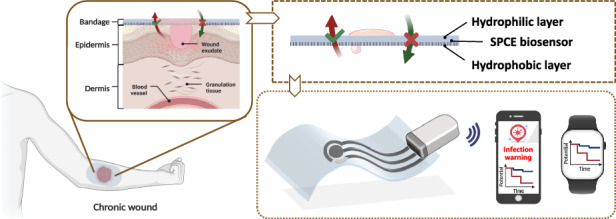

## Introduction

Chronic wounds with impaired healing ability are of significant concern in clinical care, as they affect the quality of life and incur high treatment costs^1^. Recent data indicates that more than 40 million people worldwide suffer from chronic wounds, which cause long-term physical and mental suffering and contribute to rapidly growing global economic burdens^2,3^. Therefore, it is crucial to implement effective strategies for managing chronic wounds to increase life expectancies and alleviate the burden on health-care systems.

In recent years, rapid developments have taken place regarding wound dressings and bandages, offering new solutions for advanced wound care and facilitating rapid wound healing. For example, traditional gauze dressings have been developed as hydrogel dressings that are capable of controlled drug release. These dressings enable efficient drug delivery while reducing the need for frequent changes^4,5^. Similarly, wearable wound dressings that can provide electrical stimulation to chronic wounds offer novel strategies for accelerated wound recovery^6^. Moreover, a myriad of approaches that utilize antibiotic drugs^7^, near-infrared photothermal therapy^8^, temperature-activated electromechanical synergistic therapy^9^, and other techniques have emerged to prevent wound infections and facilitate wound healing, holding great promise in assisting in the treatment of chronic wounds. However, these wound dressings with drug release or stimulation therapy capabilities, which do not require frequent dressing changes, share a common limitation: the inability to clean and drain wound exudate. As a result, wound exudate accumulates on wound surfaces for extended periods, which is an overlooked but prevalent problem hindering the wound healing process^10^.

Thus, self-pumping dressings have been developed to drain excessive biofluids around wounds, facilitating wound cleaning and drying and accelerating the wound healing process. For instance, Lianxin et al. introduced the first self-pumping dressing by electrospinning a hydrophobic nanofiber array onto a hydrophilic microfiber network, enabling the unidirectional drainage of excessive biofluids away from wounds^11^. Zhihong and colleagues subsequently developed a single-layer Janus membrane through ammonia alkali etching, superhydrophobic modification, and a single-face hydrophilization treatment on copper mesh. This membrane enhanced the breathability of the dressing and significantly reduced the critical breakthrough pressure for unidirectional water transport^12^. Furthermore, self-pumping dressings based on biocompatible materials have been extensively developed through physical modifications, chemical modifications, and microstructure alteration to effectively manage excess wound exudate and promote faster wound healing^13^. However, these self-pumping dressings have limited functionality, as they are not integrated with sensing modules, making it challenging to assess the wound healing status, thus hindering timely wound management and appropriate dressing changes.

Among the various physiological and biochemical indicators, such as temperature, moisture, pH, oxygen, uric acid, and bacterial load, the pH environment of wounds is among the most important factors that indicate wound status^14^. Specifically, alkaline microenvironments with a pH range of 7.0–9.0 are often associated with unhealed chronic wounds or infected trauma, whereas an acidic environment with a pH range of 4.5–5.0 indicates regeneration of the epidermal layer and wound healing^15^. Most recently, Xinming et al. developed a multifunctional Janus membrane integrated with phenol red nanoparticles to unidirectionally drain wound exudate while sensing the wound pH levels^16^. Similarly, a pH-responsive cellulose-based Janus nonwoven dressing with unidirectional liquid drainage capability was proposed for chronic wound management by Zhan et al.^17^. Although both of these wound dressings can estimate the wound pH environment through in situ colorimetry, the Janus membrane-based pH biosensor was designed for single-use applications and lacked the ability to dynamically sense the pH levels around the wound. Therefore, there is an urgent need to develop smart bandages that can dynamically reflect the wound status and continuously drain excessive wound exudate to facilitate advanced wound management.

Herein, we developed a flexible, wearable, robust, and low-cost smart bandage for the unidirectional drainage of wound exudate and dynamic monitoring of the wound status, aiming to facilitate advanced wound care. A bilayer membrane with asymmetric wettability that enables continuous drainage of wound exudate from the wound surface while preventing backflow is referred to as “liquid diode”. The developed liquid diode, consisting of an ultrahydrophilic polyether sulfone (PES) membrane and a hydrophobic thermoplastic polyurethane (TPU) membrane, is integrated with a 3D polyaniline mesh (M-PANI)-based electrochemical sensing interface to simultaneously clean the wound surface and dynamically monitor the wound pH environment. The M-PANI sensing interface, crosslinked with phytic acid, demonstrates excellent sensitivity, selectivity, and long-term stability, making it suitable for dynamic pH sensing and providing real-time information on wound status without the need for frequent bandage changes. To evaluate the practicality of this smart bandage, we conducted fluid and electrochemical tests using standard pH solutions and simulated skin with various pH values. The results confirmed the suitability of this smart bandage for advanced wound care and monitoring. In the future, we envision the integration of the presented smart bandage with additional sensing and treatment modules, such as drug delivery systems, electrical stimulus modules, and multimodal sensing modules, thereby building a theranostic platform for home-based wound care.

## Results

### Principles of liquid diode-based smart bandages for wound management

**Fig. 1:**
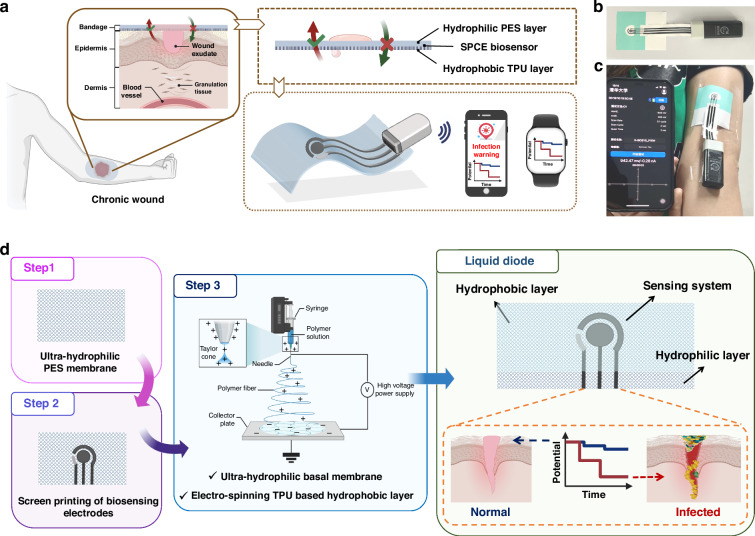
Schematic illustration of the design and fabrication of the integrated smart bandage. **a** Advanced wound care through real-time exudate draining and dynamic microenvironment sensing using the proposed liquid diode-based smart bandage. Physical images of the integrated smart bandage before (**b**) and during (**c**) application. **d** Schematic diagram depicting the detailed processes involved in the fabrication of the integrated smart bandage, including surface modification of the PES membrane through UV photoinitiated grafting, screen printing of the three-electrode system, and electrospinning of the TPU membrane. PES polyether sulfone, TPU thermoplastic polyurethane. SPCE screen-printed carbon electrode

The developed smart bandage utilizes a liquid diode and an ultrasensitive M-PANI based biosensor to achieve advanced chronic wound care and monitoring. Figure 1a illustrates the configuration of the smart bandage, which is composed of a bilayer membrane with asymmetric wettability, an integrated screen-printed carbon electrode, and a portable electrochemical workstation. Images of the real system before and during application can be found in Fig. 1b, c, and the structure of the bandage can be customized according to the size and type of the wound. Furthermore, the fabrication process for the smart bandage is depicted in Fig. 1d, which shows the formation and layer-by-layer structure of the membrane.

On the surface of chronic wounds, bilayer membranes act as liquid diodes, facilitating the drainage of wound exudate and preventing the backflow of contaminated fluids, thus maintaining a clean and dry wound environment. As the wound exudate is drained, it passes through the interlayer of the liquid diode and comes into contact with the M-PANI-based biosensor. This enables dynamic pH sensing of the wound exudate, providing real-time information on the wound status and timely warning of infection risks. Consequently, the proposed smart bandage can prevent infections by removing internal exudate and blocking external bacteria while promptly detecting unexpected infections through in situ pH level testing of exudate without the need for frequent dressing changes.

### Morphology characterization and asymmetric wettability evaluation of the proposed liquid diode

**Fig. 2:**
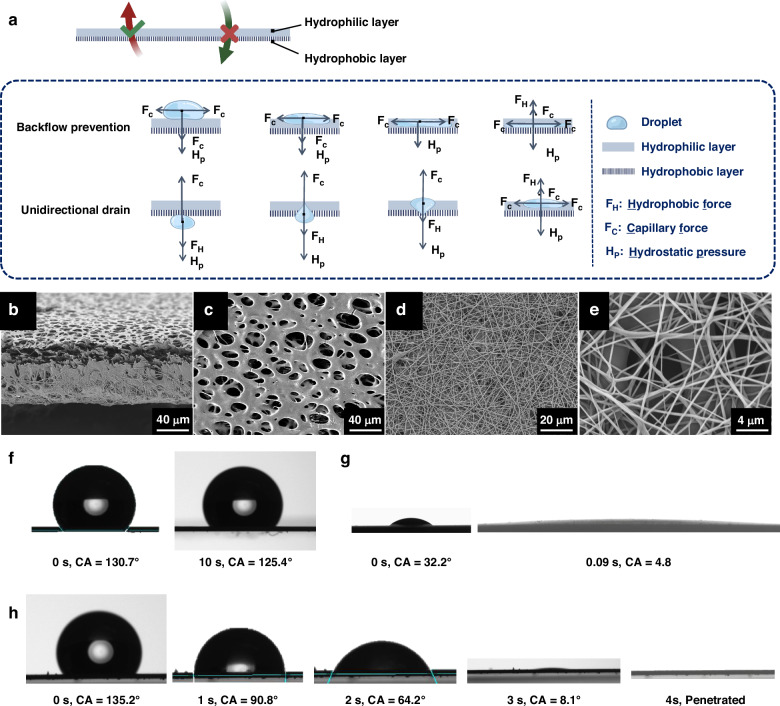
Morphology characterization and asymmetric wettability verification of the proposed liquid diode. **a** Water transport processes of the liquid diode in both the positive and reverse directions. **b** SEM images showing the cross-sectional morphology of the bilayer liquid diode at ×500 magnification, with the PES membrane on the top and the TPU membrane on the bottom. SEM images showing the surface morphology of the **c** ultrahydrophilic PES membrane at ×1000 magnification and the TPU membrane at **d** ×1000 magnification and **e** ×5000 magnification. Water contact angle measurements for the **f** hydrophobic TPU membrane, **g** ultrahydrophilic PES membrane, and **h** biolayer liquid diode

An ultrahydrophilic PES membrane was constructed for integration with a TPU membrane through electrospinning to create a bilayer membrane. The properties of this ultraphydrophilic PES membrane are compared with those of the polyethylene terephthalate (PET) membrane shown in Figs. S1–3. This bilayer membrane, known as a liquid diode, exhibits asymmetric wettability on its two sides, enabling unidirectional liquid transportation. The water transportation process in the positive direction (from the hydrophobic TPU layer to the hydrophilic PES layer), and prevention of backflow in the reverse direction (from the hydrophilic PES layer to the hydrophobic TPU layer) are depicted in Fig. 2a. Imbalanced forces, including the hydrostatic force (*F*_H_), capillary force (*F*_C_), and hydrophobic force (*H*_P_), contribute to the opposite wettability on the two sides of the liquid diode. Specifically, in the positive direction, droplets can penetrate the hydrophobic layer and reach the hydrophilic layer because of the hydrostatic force and capillary force. Conversely, in the reverse direction, droplets are absorbed by the hydrophilic layer, and the hydrostatic pressure gradually decreases, impeding their infiltration into the hydrophobic layer. The rate of droplets that pass through the liquid diode can be estimated using the Laplace equation between the hydrophilic and hydrophobic layers (Materials and Methods, Eq. 1).

Figure 2b–e display the cross-sectional and surface morphology of the prepared liquid diode. The thickness of the TPU textile layer is determined by the electrospinning time and controls the rate of droplet passage and gas permeability. With an increase in the thickness of the TPU layer, the porosity of the membrane decreases, reducing the power to propel droplet passage due to the decreased capillary force (as observed in Fig. S4). Finally, the thickness of the prepared liquid diode was optimized to be 121 μm, comprising a 120-μm hydrophilic PES layer and a 1-μm TPU textile layer (Fig. 2b). The ultrahydrophilic porous PES layer (Fig. 2c and S2–4) and ultrathin TPU layer (Fig. 2d, e) ensure the breathability of the liquid diode, whereas the asymmetric wettability of each layer enables unidirectional water transport, as demonstrated in Fig. 2f–h and Video 1. Consequently, a 3-μL droplet penetrated the prepared liquid diode in 4 s in the positive direction, whereas adsorbed by the hydrophilic layer if it was transported in the reverse direction, thereby satisfying the need for a smart bandage to provide dynamic wound exudate drainage and external shielding.

### Integration and characterization of the electrochemical sensing module on the proposed liquid diode

**Fig. 3:**
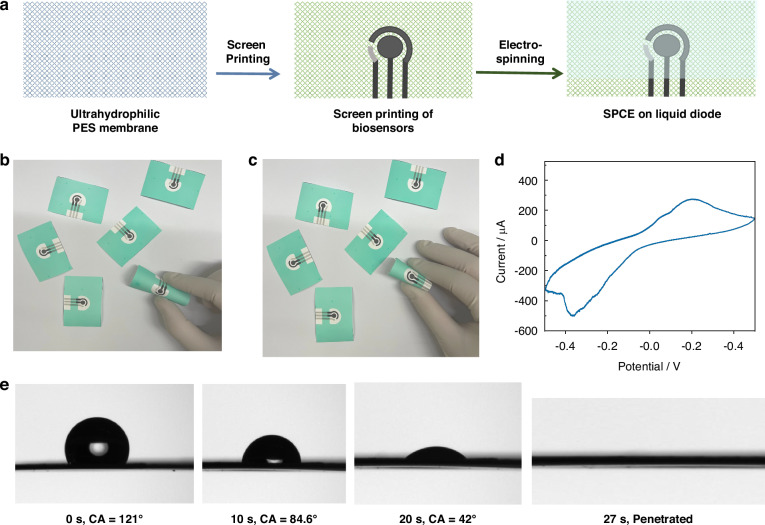
Integration of the three-electrode system on the proposed liquid diode. **a** Schematic diagram illustrating the step-by-step processes involved in the integration of the three-electrode system on the proposed liquid diode. Physical images of the integrated liquid diode **b**, **c** before and after bending in different directions. **d** Cyclic voltammetry response of the electrodes on the proposed liquid diode. **e** Dynamic water contact angles of the liquid diode after screen printing of the three-electrode system. The electrochemical tests were conducted with a voltage range from −0.2 V to 0.6 V after spiking 40 μL of 0.1 M KCl solution containing 1 mM [Fe(CN)_6_]^3−/4−^

The electrochemical sensing module for pH monitoring consists of a three-electrode system, including a working electrode, a counter electrode, and a reference electrode. The biosensor was screen-printed on an ultrahydrophilic PES membrane using carbon paste, silver paste, and insulating paste. The hydrophobic TPU membrane was subsequently electrospun to prepare the liquid diode and encapsulate the biosensor. Consequently, the biosensor was fabricated in the interlayer of the liquid diode, as shown in Figs. 3a and S5. Therefore, the diffusion rate of liquid across the liquid diode determines the time required for biochemical detection with the biosensor. The integrated liquid diode with the three-electrode biosensor exhibits excellent flexibility in both the horizontal and vertical directions when subjected to stretching, bending, curling, and folding (Fig. 3b, c, video 2), while maintaining similar gas permeability before and after the integration of electrodes (Fig. S6). This ensures the adhesion of the smart bandage to human skin and promotes breathability for wound healing.

Furthermore, the electrochemical properties of the integrated biosensor were tested through cyclic voltammetry by adding one drop of potassium ferricyanide solution to the hydrophobic TPU membrane. As depicted in Fig. 3d, the integrated biosensor displayed a clear redox curve with symmetrical oxidation and reduction voltages during the droplet transportation process, indicating the ability of the integrated liquid diode to drain and sense exudate simultaneously. Specifically, the customized hydrophilic conductive paste coating enabled successful droplet penetration while prolonging the penetration time to 27 s, ensuring stable electrochemical testing.

### Design and morphology characterization of the M-PANI/SPCE

**Fig. 4:**
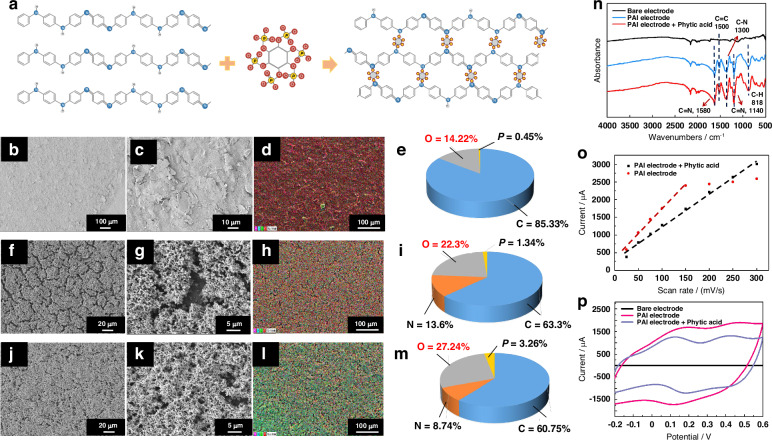
Design and property characterization of the M-PANI/SPCE. **a** Principle for the formation of a polyaniline network mediated by phytic acid. SEM images of bare SPCE after **b** 100 and **c** 1000 modifications, respectively. SEM images of **f**, **g** L-PANI/SPCE and **j**, **k** M-PANI/SPCE after 500 and 1000 modifications, respectively. EDS images of **d** bare SPCE, **h** L-PANI/SPCE, and **l** M-PANI/SPCE at ×100 magnification. Elemental analysis of **e** bare SPCE, **i** L-PANI/SPCE, and **m** M-PANI/SPCE. **n** FTIR spectra of the bare SPCE, L-PANI/SPCE and M-PANI/SPCE. **o** Plot of the peak oxidation currents versus scan rates for the L-PANI/SPCE and M-PANI/SPCE. The peak oxidation current increased linearly with increasing scan rate. **p** Cyclic voltammetry response of the L-PANI/SPCE and M-PANI/SPCE in 0.5 M H_2_SO_4_ at a scan rate of 100 mV/s

To endow the integrated liquid diode with an pH-sensing function, an ultrasensitive pH-sensing interface based on the 3D mesh PANI network was constructed on the screen-printed three-electrode biosensor. In brief, PANI has been well demonstrated to accurately sense pH levels^18^. However, the gaps between the long chains of linear PANI hinder electron transfer, thus limiting the conductivity of PANI and reducing the sensitivity of current pH sensors. Therefore, phytic acid, with six phosphate groups on one molecule that can interact with more than one polyaniline chain, provides a promising approach for constructing the PANI mesh network to increase the sensitivity and stability of pH biosensors (Fig. 4a).

As shown in Figure 4b–m, the PANI interface was successfully immobilized on the working electrode of the SPCE through the electrodeposition process by optimizing the deposition potential and time, as demonstrated in Fig. S7. For linear PANI (L-PANI) prepared in the deposition solution without phytic acid crosslinkers, the microstructure clearly exhibited cracks (Fig. 4f, g). In comparison, the morphology of M-PANI connected by phytic acid shows a well-established 3D interface composed of the PANI network (Fig. 4j–l). Additionally, the noticeable increase in the oxygen and nitrogen contents ensures the coverage of PANI, whereas the higher proportions of phosphorus and oxygen confirm the introduction of phytic acid (Fig. 4e, i, m). Furthermore, the infrared spectra of the SPCE, L-PANI/SPCE, and M-PANI/SPCE are shown in Fig. 4n. The nearly identical wavenumbers of the characteristic peaks for L-PANI/SPCE and M-PANI/SPCE indicate that the crosslinking of PANI by phytic acid relies primarily on hydrogen bonding and that the chemical structure of PANI remains unaffected.

### Reaction kinetic analysis of M-PANI/SPCE

Figure 4o shows the cyclic voltammograms of the L-PANI/SPCE and M-PANI/SPCE at different scan rates. The oxidation current increased as the scan rate increased from 25 to 300 mV/s with a 25 mV/s interval (Fig. S8). As shown in Fig. 4o, the peak oxidation current of both the L-PANI/SPCE and the M-PANI/SPCE linearly increased (*R*^2^ > 0.99) as the scan rate increased. However, compared with L-PANI, the M-PANI/SPCE demonstrated linearity over a wider scan rate range. According to the Randles–Sevcik equation, the reaction mechanism of pH with PANI-based electrodes is surface adsorption-controlled^3^. This indicates that the hydrogen ions first attach to the surface of the PANI interface, initiating the protonation of nitrogen atoms in PANI, and then, the redox reaction begins to facilitate electron transfer (Note S1). The interconnected M-PANI network increased the scan rate response range and promoted electron transfer (Fig. 4p), indicating the enhanced detection performance of the M-PANI/SPCE for pH sensing.

### Electrochemical performance characterization of the M-PANI/SPCE for pH sensing

#### Sensitivity and detection range assessment of the M-PANI/SPCE

**Fig. 5:**
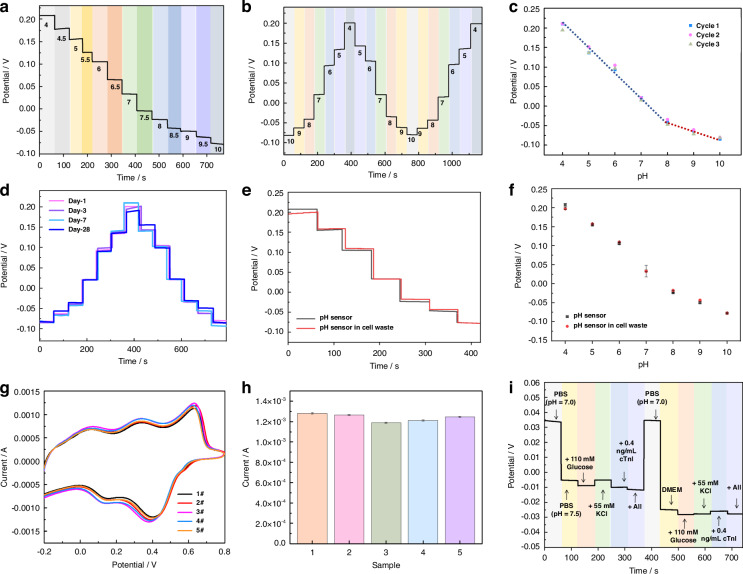
Performance characterization of the M-PANI/SPCE for pH sensing. **a** OCPT responses of M-PANI/SPCE with increasing pH from 4.0 to 10.0. **b** Reversibility evaluation of M-PANI/SPCE over the pH range from 4.0 to 10.0. **c** Linear curve fitting of the open-circuit potential versus pH ranging from 4.0 to 10.0 for the M-PANI/SPCE. **d** Stability evaluation of M-PANI/SPCE during storage. The biosensors were retrieved on Day 1, Day 3, Day 7, and Day 28 from a 4°C refrigerator, and their performance was investigated using OPCT measurements with PBS at different pH values. **e** Stability evaluation of the M-PANI/SPCE under continuous use. **f** OCPT responses of the M-PANI/SPCE at different pH values ranging from 4.0 to 10.0 after 0 h and 48 h of use. **g** Cyclic voltammetry curves and **h** histogram of the peak oxidation currents, illustrating the batch-to-batch variations in the M-PANI/SPCE. **i** Selectivity test of the M-PANI/SPCE by adding glucose at 110 mM, KCl at 55 mM, cTnI at 0.4 ng/mL, and a mixture of the abovementioned interferents in PBS at pH 7.5 and DMEM at pH 8.0. The error bars represent the standard errors of the means (*N* = 3)

The OCPT curves were tested once and in a continuous loop under different pH values of PBS, as presented in Fig. 5a, b and Fig. S9. Figure 5c shows the linear relationship between the potential signal and the pH of PBS. The linear range is divided into two sections because of the different sensitivities of M-PANI under acidic and alkaline environments. The corresponding linear equations are P$({\rm{V}})=-0.0615{{X}}_{{pH}}+0.4537$, *R*^2^ > 0.993 for acidic and neutral solutions (pH 4.0 to 8.0) and ${\rm{P}}({\rm{V}})=-0.02045{{X}}_{{pH}}+0.1209$, *R*^2^ > 0.988 for alkaline solutions (pH 8.0 to 10.0). According to the measured results, a wide detection range from pH 4.0 to 10.0 was achieved, and a high sensitivity of up to 61.5 mV/pH was obtained for the M-PANI/SPCE. This pH biosensor surpasses reported methods that either have limited detection ranges or exhibit relatively low sensitivities (Table 1).

**Table 1:** Comparison of the functions and properties of the proposed liquid diode-based smart bandage with those of other reported wound bandages for wound pH sensing

Sensing interface	Methods	Detection range	Sensitivity	Substrate	Functions	Ref.
IrOx/PEDOT:PSS	Amperometry	pH 6–9	59 μA/pH	Gauze	In situ wound pH sensing	^22^
PNIPAM/Cy7	Near-infrared fluorescence	pH 4–8	8.4 ratios/pH	HEMA film	Dynamic wound pH monitoring	^23^
PCL/curcumin	Colorimetry	pH 6.0–9.0	——	PCL	In situ wound pH sensing	^24^
Alginate/DelDerm	Colorimetry	pH 4–9	——	Commercial GelDerm	Drug delivery; In situ pH sensing	^25^
PAI/CMC/CQDs	Colorimetry	pH 5–8	0.067 ratios/pH	CMC hydrogel	Photothermal antibacterial; In situ wound pH sensing	^26^
PANI/Nafion	Potentiometry	pH 4–7	20 mV/pH	PI	Prevention of microbial infection; In situ wound pH sensing	^27^
DMAEA/PEGA	Potentiometry	pH 4–7	30 mV/pH	PEGA	Antibacterial; In situ wound pH sensing	^28^
M-PANI/PES-TPU	Potentiometry	pH 4.0–10.0	61.5 mV/pH	PES-TPU liquid diode	Wound exudate drainage; Wound protection; In situ dynamic pH sensing	This work

*PEDOT:PSS* poly(3,4-ethylenedioxythiophene):poly(styrene sulfonate), *PNIPAM* 2-hydroxyethyl methacrylate, *HEMA* 2-hydroxyethyl methacrylate hydrogel film, *PCL* polycaprolactone, *PAI* polyvinyl alcohol-iodine, *CMC* sodium carboxymethyl cellulose, *CQD* carbamino quantum dot, *PI* polyimide, *DMAEA* 2-(diethylamino)ethyl acrylate, *PEGA* poly(ethylene glycol)methyl ether acrylate

#### Stability evaluation of the M-PANI/SPCE

The long-term stability of the M-PANI/SPCE was assessed for both storage and dynamic monitoring, aiming to evaluate its potential in practical applications. As shown in Figs. 5d and S10, the performance of the M-PANI/SPCE remained largely unchanged after 28 days of storage in a refrigerator at 4 °C. The OCPT response in PBS at different pH values on the 1st, 3rd, 7th, 14th and 28th days varied by only 4.8%, indicating the excellent stability and prolonged working time of the M-PANI/SPCE for at least 28 days. Furthermore, the OCPT responses were collected before and after immersing the M-PANI/SPCE in cell waste fluid from human skin fibroblasts (HSFs) for 48 h, as depicted in Fig. 5e, f. The fitted linear curves for the OCPT responses before and after 48 hours of soaking, which are directly correlated with the sensitivity of the biosensor, nearly overlapped, suggesting the potential of the M-PANI/SPCE for long-term dynamic monitoring.

#### Reproducibility verification of M-PANI/SPCE

M-PANI/SPCEs were fabricated using the same preparation processes, five of which were randomly selected for measurement in a 0.5 M H_2_SO_4_ solution, as shown in Fig. 5g, h). The results indicate that the relative standard deviation (RSD) between the electrodes was 3.1%, indicating that the difference between devices is negligible, which makes mass production of the M-PANI/SPCE highly promising.

#### Selectivity evaluation of the M-PANI/SPCE

The selectivity of the prepared M-PANI/SPCE was studied by introducing H^+^ and other interferents, including metabolites such as glucose, ions such as K^+^, proteins such as cardiac troponin I (cTnI), and a mixture of all these interferents, into PBS (pH = 7.0). Figure 5i shows that the potential response of the M-PANI/SPCE to pH is nearly 10 times greater than that to the interferents, although the interferent concentrations were 10 times greater than their highest physiological concentrations. The negligible effect of interferents on the biosensor indicates that the M-PANI/SPCE is feasible for pH monitoring in the presence of other ions and biomolecules in wound exudate, demonstrating its potential for accurate and specific pH monitoring in practical applications.

### Immobilization and characterization of the M-PANI-based biosensor on an integrated liquid diode

**Fig. 6:**
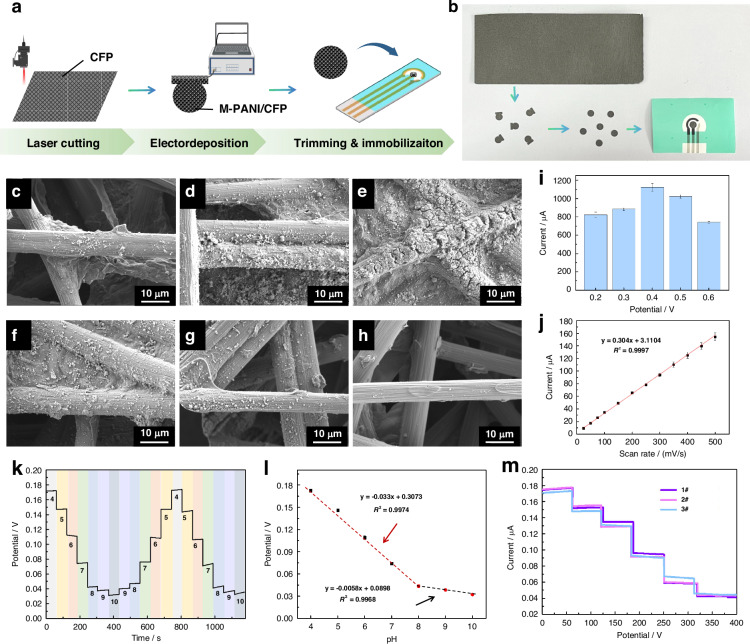
Immobilization and characterization of M-PANI/CFP on an integrated liquid diode. **a** Schematic diagram of the fabrication processes for immobilizing M-PANI/CFP on the integrated smart bandage, including laser cutting, electrodeposition, trimming and immobilization. **b** Physical images of M-PANI/CFP before and after being immobilized on the integrated liquid diode. SEM images of M-PANI/CFP at different electrodeposition potentials: **c** 0.2 V, **d** 0.3 V, **e** 0.4 V, **f** 0.5 V, **g** 0.6 V, and **h** 0.7 V. **i** Histogram of the peak oxidation currents of M-PANI/CFP at different electrodeposition potentials. **j** Plot of the peak oxidation current versus the scan rate of the M-PANI-modified smart bandages. The peak oxidation current increased linearly as the scan rate increased. **k** Reversibility evaluation of the M-PANI-modified smart bandage over a pH range from 4.0 to 10.0 on simulated skin. **l** Linear curve fitting of the open-circuit potential versus pH values ranging from 4.0 to 10.0 for the M-PANI-modified smart bandages. **m** OCPT response of the peak oxidation current, illustrating the batch-to-batch variations in the M-PANI-modified smart bandage. The error bars represent the standard errors of the means (*N* = 3). CFP carbon fiber paper, M-PANI mesh polyaniline

To facilitate the integration of the M-PANI-based biosensor with the liquid diode, the proposed 3D M-PANI network was transferred onto a portable carbon fiber paper (CFP) electrode. The transfer process was similar to the fabrication of the M-PANI/SPCE through electrodeposition, as shown in Fig. S11. The prepared M-PANI/CFP electrode was subsequently trimmed and immobilized on an integrated liquid diode to obtain a smart bandage, as depicted in Fig. 6a, b.

#### Morphological characterization of M-PANI/CFP

The microstructures and elemental compositions of PANI immobilized on the surface of CFP at different deposition potentials are presented in Fig. 6c–h and Figs. S12 and 13. The deposition efficiency initially increases and then decreases as the deposition potential increases from 0.2 V to 0.6 V. Specifically, at low deposition potentials, PANI forms scattered dot-like structures on the sensing interface. As the potential gradually increased, the scattered PANI units progressively connected, resulting in the formation of a complete 3D M-PANI structure at 0.4 V. As the deposition potential subsequently increased, the mesh structure became disrupted, and the PANI membrane on the interface surface returned to a dispersed state. Therefore, 0.4 V was determined to be the optimal potential for the formation of the M-PANI/CFP interface. This conclusion is further supported by the efficiency of electron transfer, as evidenced by the peak oxidation current in cyclic voltammetry measured in 0.5 M H_2_SO_4_, as shown in Fig. 6i and Fig. S14.

#### Electrochemical property assessment of the smart bandage

The reaction mechanism of the M-PANI/CFP for pH sensing is the same as that of the M-PANI/SPCE, which is surface adsorption-controlled. This is supported by the linear relationship observed between the scan rate and oxidation current, as depicted in Figs. 6j and S15. Furthermore, the M-PANI/CFP was trimmed and immobilized on an integrated liquid diode to construct a smart bandage, enabling dynamic pH monitoring with wound exudate extrusions on the wound surface. The sensitivity and consistency of the smart bandage for dynamic monitoring in acidic and alkaline solutions (ranging from pH 4.0 to 10.0) were tested on the simulated skin in a continuous loop, as shown in Fig. 6k, l, thereby demonstrating the practicability of the proposed smart bandage for wound status monitoring. Similarly, the batch-to-batch variations of the smart bandage were evaluated using OCPT tests on the simulated skin (Fig. 6m), verifying the reproducibility of the integrated system for advanced wound care and monitoring.

### Validation of the proposed smart bandage in preclinical wound models

**Fig. 7:**
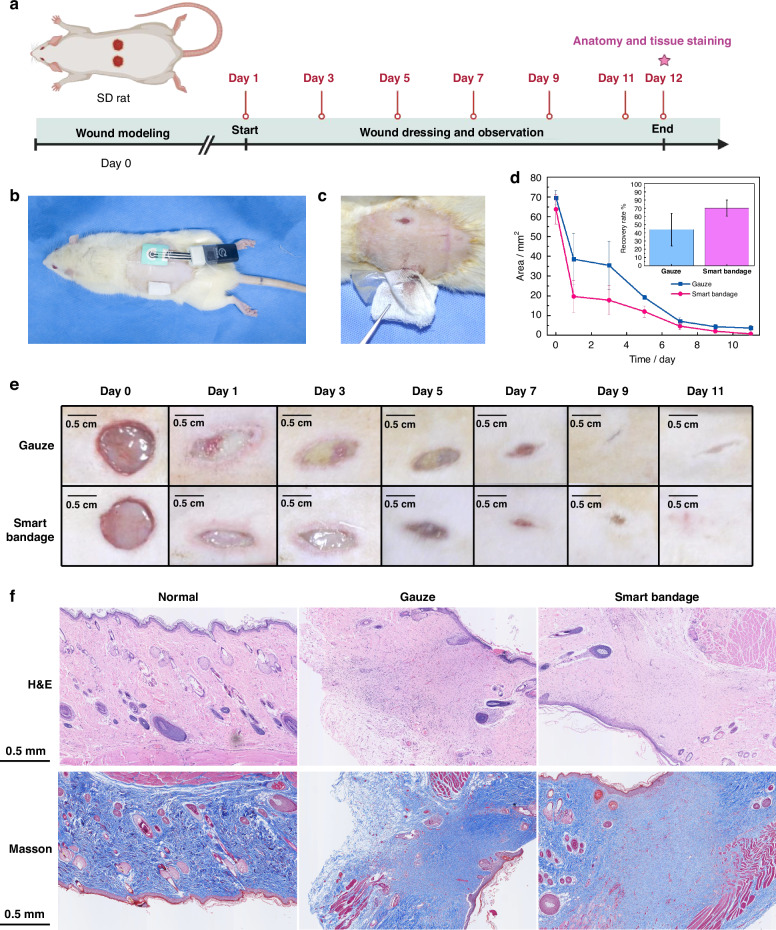
Preclinical animal test of the smart bandage. **a** Diagram of the animal experiment workflow from Day 0 to Day 12. **b** Physical images displaying the application of the smart bandage on rats. **c** Physical images captured during the dressing change. The upper wound is covered by the proposed smart bandage without adhesion, whereas the lower wound is covered by gauze, showing evident adhesion during removal. **d** The areas of the circular wounds on each side of each rat (*N* = 3). The insert shows the recovery rate of the circular wound on the first day. **e** A series of images showing the changes in wound area over time for the gauze and smart bandage groups. **f** Representative cross-sectional histological images of skin tissues harvested from normal skin, skin recovered with traditional gauze and the proposed smart bandage after 11 days. The upper images are H&E stained, and the lower images are Masson stained. Scale bar = 0.5 mm

To assess the improvements in biocompatibility and recovery with the proposed smart bandage, the wound healing performance was investigated on circular full-thickness skin wounds (0.8 cm in diameter) in rats. The process of the animal experiment is depicted in Fig. 7a. In detail, three rats were utilized for the experiment, where circular wounds were created on the left and right backs of each rat. Gauze and the proposed smart bandage were employed on the left and right wounds of each rat, respectively (Fig. 7b). With the smart bandage of asymmetric wettability, the accumulation of excessive exudate on the wound and subsequent adhesion of the wound dressing to the wound were prevented, as illustrated in Fig. 7c. In comparison, evident adhesion was observed in the gauze group. As a result, the secondary trauma caused by frequent dressing replacement can be significantly reduced, leading to an improved wound recovery rate.

Figure. 7d, e presents the qualitative and quantitative results obtained during the wound healing process, confirming that the proposed smart bandage can accelerate wound healing compared to conventional gauze. This effect is particularly evident during the initial stages of healing, where the recovery rate significantly increased from 43.9% to 70.2%, largely due to the rapid drainage of excessive wound exudate. Drainage of wound exudate not only helps prevent wound infection but also provides a more suitable environment for wound healing, resulting in an increased recovery rate and tissue growth.

Figure 7f shows the results of hematoxylin‒eosin (H&E) staining and Masson’s trichrome staining of normal skin and recovered skin with traditional gauze and the proposed smart bandage. First, the dermal thickness of the wound tissues after 11 days of healing with the assistance of traditional gauze and the proposed smart bandage was consistent with that of normal skin tissues. In addition, extensive blue-stained collagen fibers (>75%) were visible in the dermal layer and subcutaneous tissue in both the gauze and smart bandage groups, confirming the gradual restoration of the skin structure at the wound site. These findings suggest that the proposed smart bandage accelerates wound closure without interfering with the normal repair of the dermal layer.

Moreover, significant neovascularization was observed in the subcutaneous area of the skin at the wound sites, which was assisted by both gauze and the smart bandage. The number of neovascularization groups, crucial for tissue regeneration, development, and repair, ranged from 4 to 7 in the skin recovered with gauze. In contrast, enhanced neovascularization (>7 groups) was observed in the recovered skin with smart bandages, accompanied by fibroblast structures. The increased number of newly formed vessels indicates the rapid recovery and repair of the skin’s function and structure at the wound site after the use of the proposed smart bandage.

Overall, a flexible liquid diode with asymmetric wettability was proposed for integration with a biosensing module for advanced wound care and monitoring. The liquid diode facilitated unidirectional transport of droplets, allowing for efficient drainage of wound exudate from wound surfaces. The pH levels of the wound exudate were then tested during the process of droplet passage. Moreover, our developed M-PANI-based biosensors demonstrated exceptional performance in terms of sensitivity, stability, reproducibility, and selectivity for ultrasensitive pH sensing. This biosensor can be seamlessly integrated with a liquid diode, and its practical potential has been verified on simulated skin and in rat models, thus making it highly promising for pH monitoring in wound management applications.

## Discussion

In this work, we successfully developed a flexible, wearable, robust, and low-cost smart bandage for unidirectional wound exudate drainage and dynamic wound status monitoring, aiming to facilitate advanced wound care. To the best of our knowledge, the smart bandage we have developed, which consists of an ultrasensitive pH biosensor integrated with a liquid diode, is the first of its kind to achieve the dual functionality of wound exudate cleaning and monitoring. This breakthrough enables accurate, specific, and dynamic pH monitoring of wound surfaces, making it a feasible and promising approach for wound care (Table 1).

Specifically, a liquid diode based on asymmetric wettability allows the penetration of wound exudate in the positive direction from the hydrophobic layer to the hydrophilic layer, while blocking backflow and preventing the passage of fluid in the reverse direction. The prepared ultrathin liquid diode, composed of a biocompatible PES membrane and TPU membrane, offers excellent flexibility, breathability, and the ability to transport water unidirectionally, ensuring effective wound exudate cleaning and isolation of external fluids. The animal experiments further verified the excellent biocompatibility of the proposed smart bandage and its ability to accelerate wound healing effectively compared with that of conventional gauze.

Additionally, the 3D M-PANI-based biosensor utilized phytic acid, which has six phosphate groups on one molecule, to crosslink with the linear PANI material to construct a mesh network for pH sensing. The mesh network facilitates efficient electron transfer, endowing the sensor with high sensitivity, excellent selectivity and high stability for dynamic pH monitoring. Specifically, the M-PANI-based biosensor exhibited outstanding performance, with a wide linear range from pH 4.0 to 10.0 and a high sensitivity of 61.5 mV/pH, which covers the entire range of pH values for normal wounds (pH 4.5–5.0) and infected wounds (pH 7.0–9.0)^19^. Moreover, the M-PANI-based biosensor is ready for mass production because it is easy to fabricate via one-step electrodeposition procedures and has a negligible batch-to-batch variation of only 3.1%. Furthermore, the biosensor demonstrated long-term stability, with a variation of only 4.8% in the OCPT response after 28 days of storage. The fitted linear curves before and after 48 h of dynamic testing in a cell culture medium with HSF cells overlapped, indicating that the proposed biosensor has a long working life and can meet the practical demands for wearable wound care without the need for frequent bandage changes.

Finally, both the liquid diode and the M-PANI-based biosensor have the potential to be expanded to other applications. First, the liquid diode can be woven into gym suits and integrated with pH sensors and a series of other biosensors to enable sweat drainage and metabolite sensing, facilitating personalized fitness tracking and health management. In addition, the M-PANI-based biosensor can be designed as a point-of-care device, offering immense potential in microenvironment testing to accelerate advancements in health care, environmental sustainability, and food safety.

In conclusion, we have proposed a novel kind of smart bandage composed of a liquid diode and an ultrasensitive pH biosensor for advanced wound care. This smart bandage enables the unidirectional drainage of wound exudate for effective wound cleaning and protection, as well as the dynamic monitoring of pH levels in the exudate for wound status evaluations, which aligns with the emerging clinical demands in wound care. The construction and application of this kind of smart bandage not only facilitates enhanced monitoring, treatment and prognosis of chronic wounds, but also empowers personalized health management and fitness tracking, promoting the realization of precision medicine and smart medicine.

## Materials and methods

### Materials preparation

Ultrahydrophilic PES membranes were purchased from Membrane Solutions, LLC (Seattle, WA). Thermoplastic polyurethane (TPU, Tecoflex SG-93A) was purchased from Lubrizol Life Science Health, LLC (Brecksville, OH). Sulfuric acid (H_2_SO_4_, 98%) was purchased from Fluka (Shanghai, China). Aniline, potassium chloride (KCl), potassium ferricyanide (K_3_Fe(CN)_6_) and potassium ferrocyanide (K_4_Fe(CN)_6_) were purchased from Aladdin (Shanghai, China). Phosphate buffer solutions (PBS, pH=4.0, 4.5, 5.0, 5.5, 6.0, 6.5, 7.0, 7.5, 8.0, 8.5, 9.0, 9.5 and 10.0) and phytic acid (50 wt% in H_2_O) were obtained from Macklin Biochemical Technology Co. Ltd. (Beijing, China). Dimethyl formamide (DMF) and tetrahydrofuran (THF) were supplied by Xilong Scientific Co., Ltd. (Guangdong, China). All other chemicals used were of analytical grade without further purification. Double distilled water (ddH_2_O) was used throughout the experiments. Electrospinning was conducted by Beijing Ucalery Technology and Development Co. Ltd. (Beijing, China). Electrochemical measurements were performed using a CHI760E electrochemical workstation (Chenhua, Shanghai, China) and a BioSYS-P15E portable electrochemical workstation (Shuaxin, Shenzhen, China). Animal experiments were conducted in cooperation with WEIKE Inspection Group Co., LTD (Shenzhen, China).

### Preparation of the liquid diode

First, PES membranes were treated by ultraviolet light grafting to obtain ultrahydrophilic PES membranes, which served as the hydrophilic layer of the liquid diode. Next, TPU was dissolved in a mixed solvent of DMF and THF (v/v = 1:1) overnight to obtain a 12.5 wt% TPU electrospinning solution. The TPU solution was subsequently electrospun at 15 kV with a supply rate of 1.5 mL/h. The electrospun TPU membrane was collected onto an ultrahydrophilic PES membrane. Therefore, a Janus liquid diode with asymmetric wettability was successfully prepared. To integrate the sensing module onto the prepared liquid diode, the three-electrode system was screen-printed on the ultrahydrophilic PES membrane prior to electrospinning, allowing for the fabrication of the sensing electrode within the interlayer of the liquid diode.

The main physical forces involved in the unidirectional droplet transport of the liquid diode are the hydrostatic force (*F*_H_) and the hydrophobic force (*H*_P_), which exert opposing effects on water permeation. When these imbalanced forces attract the droplet to the interface between the hydrophobic and hydrophilic layers, the capillary force (*F*_C_) at this interface accelerates the penetration of the droplet. The capillary forces relevant to the performance of the liquid diode can be calculated via the Young–Laplace Equation^20^.

1 $${F}_{C}=\frac{\gamma }{r}\cos \theta$$

In Eq. (1), ${\rm{\gamma }}$ represents the surface tension of the liquid, *r* represents the pore radius of the membrane, and ${\rm{\theta }}$ represents the contact angle between the liquid and surface.

### Fabrication of the M-PANI-based biosensor

Phytic acid and aniline, with a molar ratio of 6:1, were added to 100 mL of a 1 M H_2_SO_4_ solution under vigorous stirring for 24 h to form a clear solution. Then, M-PANI was electrodeposited onto the working electrode by immersing the screen-printed carbon electrode (SPCE) in the deposition solution and applying a voltage of 0.6 V for 120 s to form the M-PANI/SPCE. The deposition voltage was screened from 0.4 V to 1.0 V, while the deposition time was optimized between 60 s and 240 s with a 60 s interval. For comparison, 0.1 M aniline was added to a 1 M H_2_SO_4_ solution to form L-PANI via the same electrodeposition procedure.

The preparation of the M-PANI/CFP followed similar procedures as mentioned above. Briefly, both sides of CFP were subjected to a vacuum plasma cleaning process for 45 seconds to clean and activate the surface. Then, M-PANI and L-PANI were electrodeposited on the CFP surface by immersing it in the deposition solution and applying a voltage of 0.4 V for 300 s, respectively. All the processes were conducted under ambient conditions.

### Characterization of the liquid diode

Morphological characterization of the liquid diode was carried out by scanning electron microscopy (JSM-7500F, JEOL, Japan) to study the topography and thickness of the porous PES layer, TPU nanofiber layer and bilayer liquid diode.

The wettability of each layer of the liquid diode was evaluated by measuring the water contact angles with a contour analysis system (OCA25, Dataphysics, Germany). A high-speed charge-coupled device camera (UI-2250SE-C-HQ, IDS Imaging Development Systems GmbH, Germany) was used to record the dynamic immersion process of the water. The volume of the water droplet was set at 3 μL.

The air permeability of the membranes was evaluated by calculating the rate of moisture evaporation from the centrifuge tubes over a period of 48 h. The centrifuge tubes were sealed with PES membranes, liquid diodes, and smart bandages, while fully open and fully sealed containers were used as negative and positive control groups, respectively. The initial liquid contents inside the centrifuge tubes were 10 mL.

### Morphological and structural characterization of the M-PANI-based biosensor

Scanning electron microscopy with an energy-dispersive X-ray detector (JSM-7500F, JEOL, Tokyo, Japan) was employed to observe the surface morphologies and element distributions of L-PANI and M-PANI on SPCE and CFP, respectively. Furthermore, attenuated total reflectance Fourier transform infrared spectroscopy (ATR−FTIR; Nicolet 6700, Thermo Fisher, Waltham, MA) was performed to analyze the chemical structure of M-PANI, confirming the crosslinking mechanism of phytic acid.

### Electrochemical characterization of the M-PANI-based biosensor

#### Reaction kinetic analysis of the M-PANI-based biosensor

The electrochemical performance of the L-PANI- and M-PANI-based biosensors was studied in a solution of 0.1 M KCl containing 1 mM [Fe(CN)_6_]^3−/4−^, and the cyclic voltammetry response from −0.2 V to 0.6 V was recorded. Cyclic voltammograms were recorded at various scan rates, ranging from 25 to 100 mV/s with a 25 mV/s interval and from 100 to 500 mV/s with a 50 mV/s interval. The linear relationship between the peak current of the cyclic voltammetry curve and the scan rate provides insights into the interfacial reaction kinetics of the M-PANI-based biosensor, as described by the Randles–Sevcik equation^21^.

#### Linear range measurement of M-PANI-based biosensors

The linear range and LOD of the M-PANI-based biosensor were evaluated by recording the open-circuit potential-time (OCPT) response in PBS at different pH values ranging from 10.0 to 4.0. In brief, M-PANI-based biosensors were immersed in 2 mL of PBS, and OCPT tests were carried out continuously. To facilitate the formation of a double electrode layer on the sensing interface and lower the background potential, the biosensors were submerged in the solutions for 1 minute before initiating the tests. All tests were performed at room temperature, and each experiment was repeated three times.

#### Assessment of the repeatability of M-PANI-based biosensors

M-PANI-based biosensors were tested in a continuous loop using PBS solutions that changed from alkaline to acidic conditions (from pH 10.0 to 4.0) and then from acidic to alkaline conditions (from pH 4.0 to 10.0) to evaluate the repeatability of the biosensor for dynamic testing.

To evaluate the repeatability of the biosensor for large-scale production, six randomly selected M-PANI/SPCE samples were subjected to cyclic voltammetry tests in 0.5 M H_2_SO_4_. The potential range during the cyclic voltammetry tests was between −0.2 and 0.8 V, with a scan rate of 100 mV/s. Furthermore, the OCPT response of each sample was individually measured in PBS solutions with pH values ranging from 10.0 to 4.0 to calculate the batch-to-batch variations of the M-PANI-based biosensors.

#### Stability evaluation of M-PANI-based biosensors

To evaluate the long-term stability of the M-PANI-based biosensors for storage, the biosensors were washed with DI water, dried, and stored at room temperature for 28 days. On the 1st, 3rd, 7th, 14th and 28th days, the biosensors were retrieved, and their performance was assessed through OCPT measurements. Each experiment was conducted at room temperature and repeated three times.

Furthermore, to assess the long-term stability of the M-PANI-based biosensors for dynamic testing, several M-PANI/SPCE samples were tested in PBS at different pH values ranging from 10.0 to 4.0. M-PANI/SPCE was subsequently immersed in a cell culture medium composed of HSFs for 48 h. After 48 h, the biosensor was retrieved, and the OCPT responses in various PBS solutions were recorded. Each experiment was conducted at room temperature and repeated three times.

#### Selectivity assessment of the M-PANI-based biosensor

To investigate the selectivity of the M-PANI-based biosensors, OCPT measurements were conducted by introducing various interferents into PBS at pH 7.5 and DMEM at pH 8.0. The interferents included glucose at 110 mM, KCl at 55 mM, cTnI at 0.4 ng/mL, and a mixture of these interferents. These interferents were selected because they are commonly found in human serum, and their concentrations in the tests were ten times higher than the highest concentration level observed in healthy human serum. The OCPT signals of the M-PANI-based biosensors in PBS at pH 7.0 were recorded as controls.

### Preclinical animal tests of smart bandages

Three six-week-old male Sprague‒Dawley rats were subjected to the same circular wound surgery procedure and were fed under identical conditions. Briefly, all Sprague‒Dawley rats were individually housed in separate cages in a temperature-controlled room (22°C) with a 12-hour light/12-hour dark cycle. They had free access to water and rodent feed.

The rats were anesthetized by intraperitoneal injections of Sutai 50 (5–6 mg/kg) and celazine (10–12 mg/kg). After anesthesia, the rats were fixed in the prone position, and the hair in the experimental area on the dorsal portion was shaved using electric hair clippers after the application of hair removal cream. Two full-thickness circular skin wounds (0.8 cm in diameter) were then created along the back of each rat after disinfection treatment with 0.5% iodophor.

The smart bandage and gauze were placed above the wound on the left and right sides of each rat, respectively, and the biocompatible double-sided tape was used to secure the dressing edges. The smart bandage was designed for ease of use and painless removal. The wound-healing process was documented daily.

## Supplementary information

Supplemental Material

Fluid test of the liquid diode

Mechanical toughness characterization of the liquid diode

## Data Availability

All experimental and theoretical data are available upon reasonable request from Han Wang (hanwang@tsinghua.edu.cn).
