# Supplementary material for: Chronic wound management: a liquid diode-based smart bandage with ultrasensitive pH sensing ability
Source: Microsyst Nanoeng. 2024 Dec 16;10:193. doi: 10.1038/s41378-024-00801-6 (PMC11647034; doi:10.1038/s41378-024-00801-6)
Supplement: Supplementary file 1 — Supplemental Material [file 41378_2024_801_MOESM1_ESM.docx]

SUPPORTING INFORMATION

**Chronic wound management: A liquid diode-based smart bandage with ultrasensitive pH sensing**

**Xueqi Wang ^1^, Jing Cheng**^1,2^**, Han Wang ^1^ ***

^1^School of Biomedical Engineering, Tsinghua University, Beijing, 100084, China.

^2^National Engineering Research Center for Beijing Biochip Technology, Beijing, 102206, China.

*Email: hanwang@tsinghua.edu.cn

**This PDF file includes:**

**Note S1.** Electrochemical detection mechanism of M-PANI based biosensor

**Figure S1 to S14**

**Figure S1.** Porosity assessment of PET and PES membrane.

**Figure S2.** Gas permeability of PET and PES membranes.

**Figure S3.** Water contact angle measurements of PET and PES membranes.

**Figure S4.** Morphology characterization of the proposed liquid diode with different thickness.

**Figure S5.** Screen printed carbon electrodes (SPCE) on the liquid diode.

**Figure S6.** Gas permeability of liquid diodes integrated with SPCE.

**Figure S7.** Condition optimization of electrodeposition potentials and time for M-PANI fabrication.

**Figure S8.** Reaction kinetic analysis of L-PANI/SPCE and M-PANI/SPCE.

**Figure S9.** Dynamic response of M-PANI/SPCE in various pH solutions.

**Figure S10.** Long-term stability of M-PANI/SPCE for storage.

**Figure S11.** Transfer of M-PANI based sensing interface on carbon fiber paper (CFP).

**Figure S12.** Element distribution of M-PANI/CFP fabricated at different potentials.

**Figure S13.** Oxygen percentage of M-PANI/CFP fabricated at different potentials.

**Figure S14.** Cyclic voltammetry tests of M-PANI/CFP fabricated at various potentials.

**Figure S15.** Reaction kinetic analysis of M-PANI/CFP.

**Note S1. Electrochemical detection mechanism of M-PANI based biosensor**

The developed M-PANI based biosensor relies on the electrochemical three-electrode scheme. Due to the protonation of nitrogen atoms through acid doping, the emeraldine base (EB) state of PANI can transit into an emeraldine salt (ES) state, altering the electrical performance of the sensing interface (1-2). The doping state of PANI can be influenced by acidic or basic substances present in the environment, making PANI an active material capable of converting acidic or basic environment information into electrical signals. After crosslinking PANI into M-PANI with phytic acid, the ability to transfer electrons can be further enhanced. The following chemical equation (S1) demonstrates reversible transformation of proton gaining and losing between emeraldine salt and emeraldine base in M-PANI based biosensor.


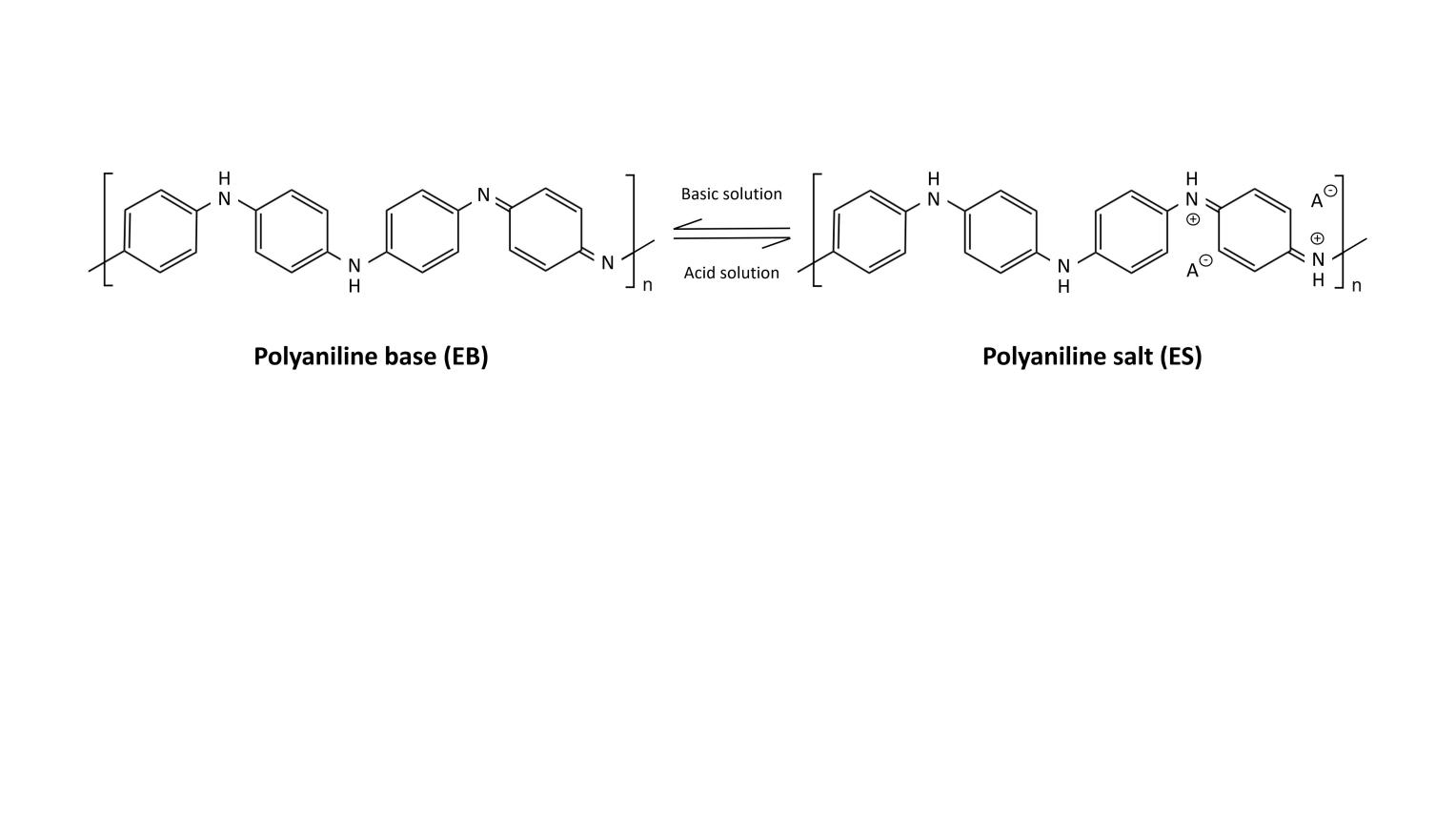
 (S1)


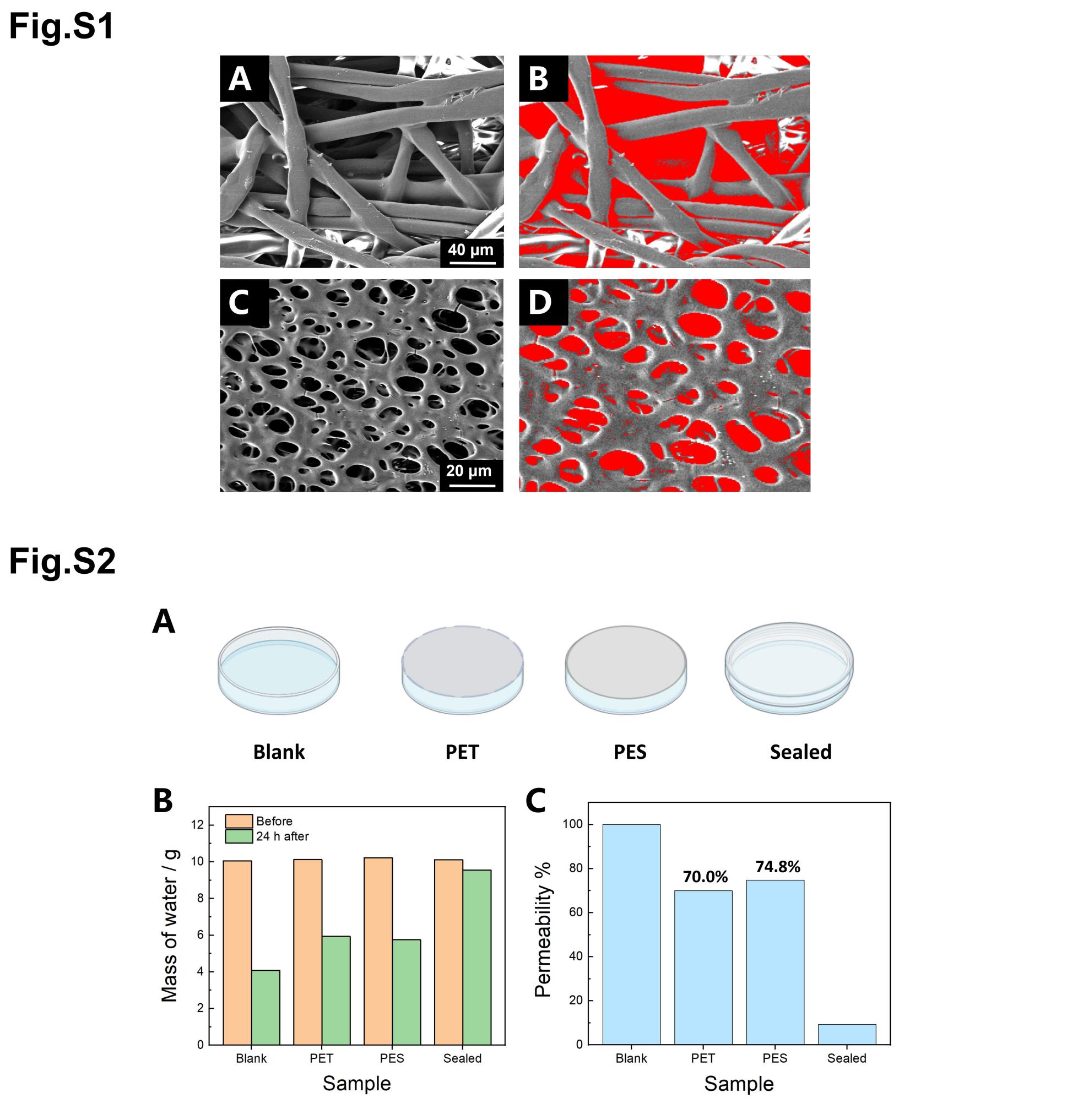


**Figure S1| Porosity assessment of polyethylene terephthalate (PET) and polyether sulfone (PES) membrane.** (A) SEM images of PET membrane at 500 times magnification. (B) SEM image of PET membrane with pores filled with red blocks. (C) SEM images of PES membrane at 1,000 times magnification. (D) SEM image of PES membrane with pores filled with red blocks. The pores in the SEM images were recognized and filled using Image-Pro Plus 6.0.


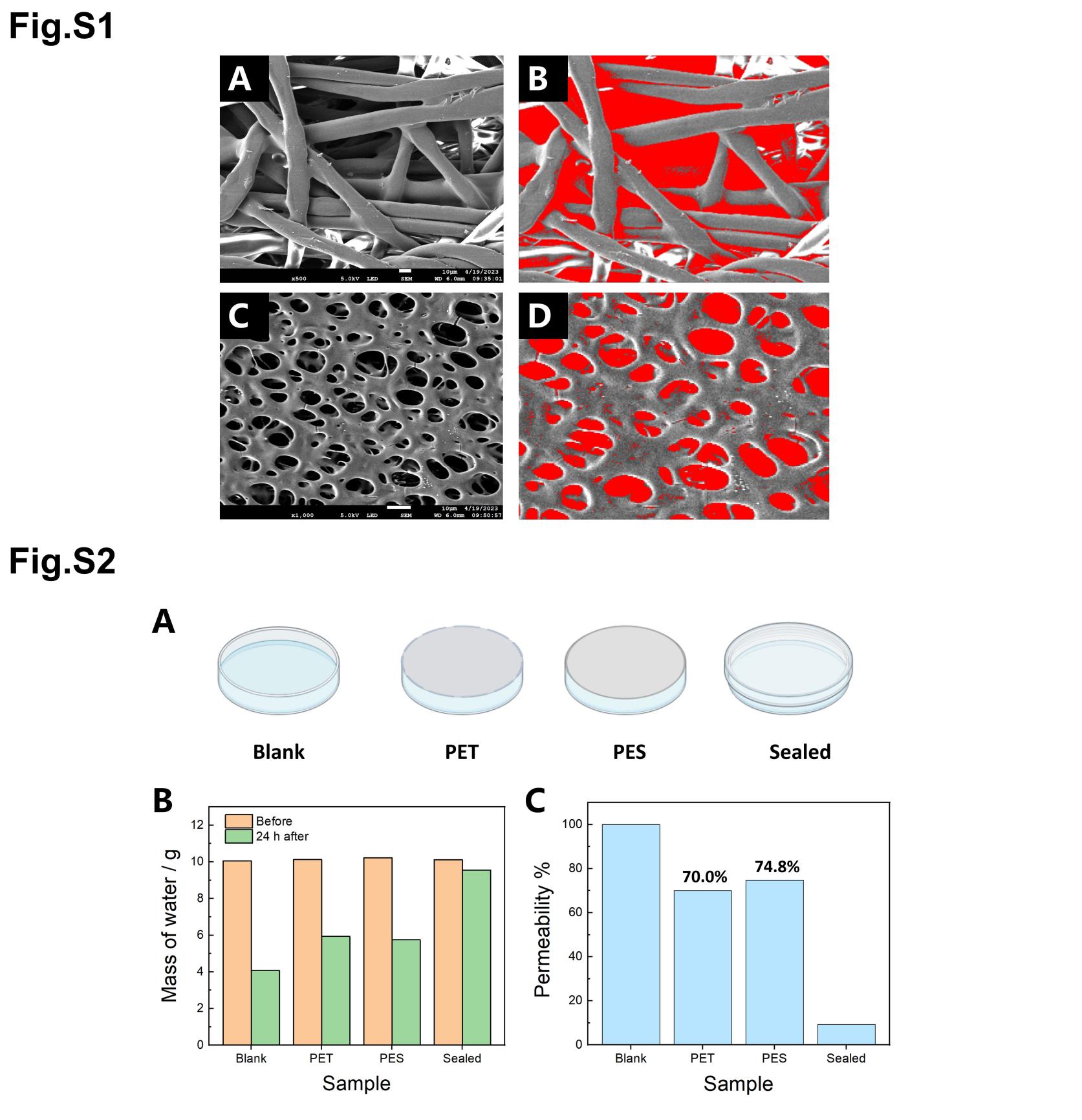


**Figure S2| Gas permeability of** **PET and PES membranes.** (A) Schematic diagram of the gas permeability test flow for the ultrahydrophilic membranes. The container of the blank group was fully open, while the container of the sealed group was completely sealed with non-breathable plastic caps. The test groups were covered by PET and PES membrane, respectively. (B) Histogram showing the water mass before and after 24 hours of evaporation for each group. (C) Histogram displaying the calculated gas permeability of each group.


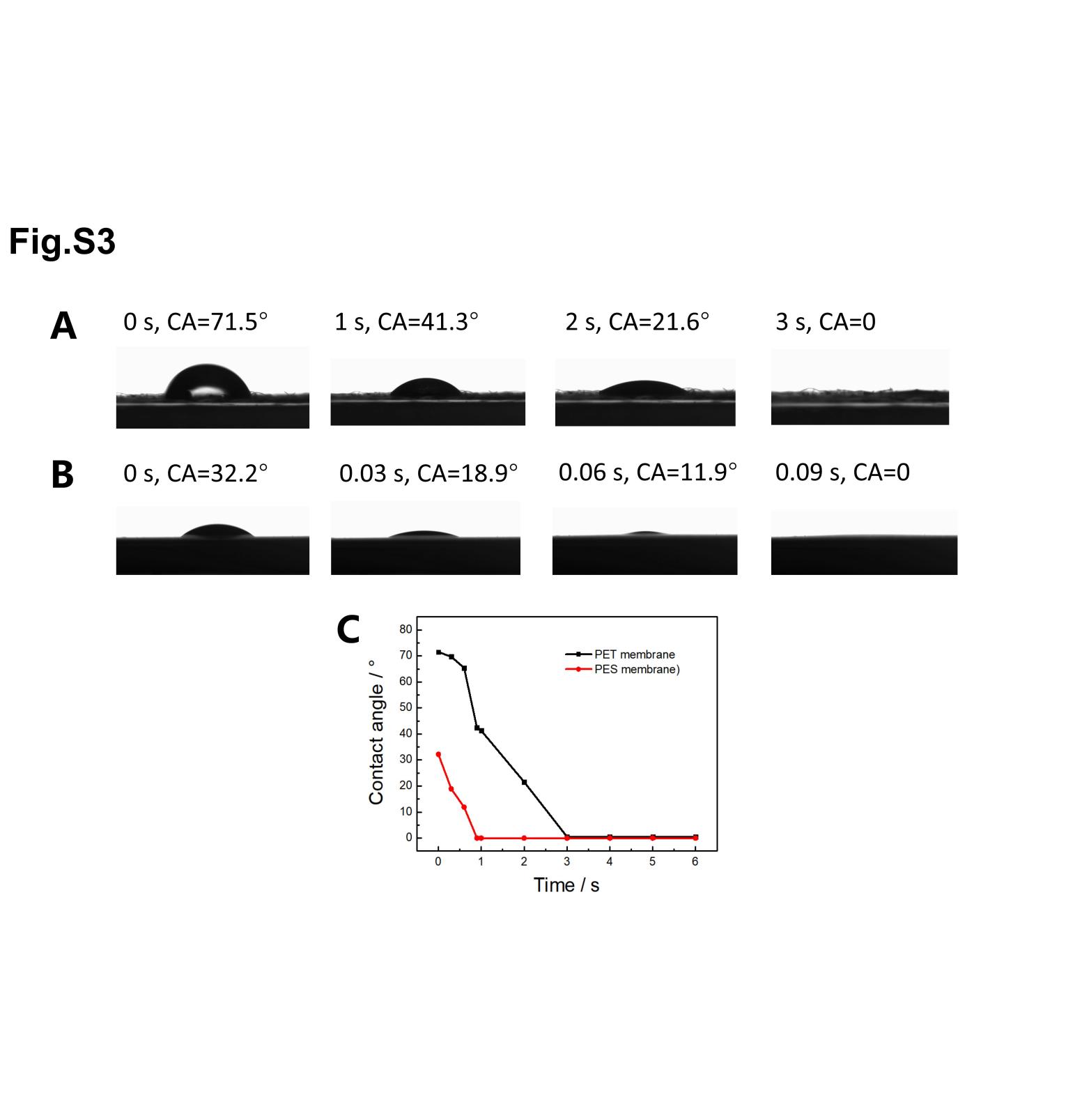


**Figure S3| Water contact angle measurements of PET and PES membranes.** Water contact angle measurements for (A) hydrophilic PET membrane and (B) ultra-hydrophilic PES membrane. (C) Dynamic water contact angles of PET and PES membranes.


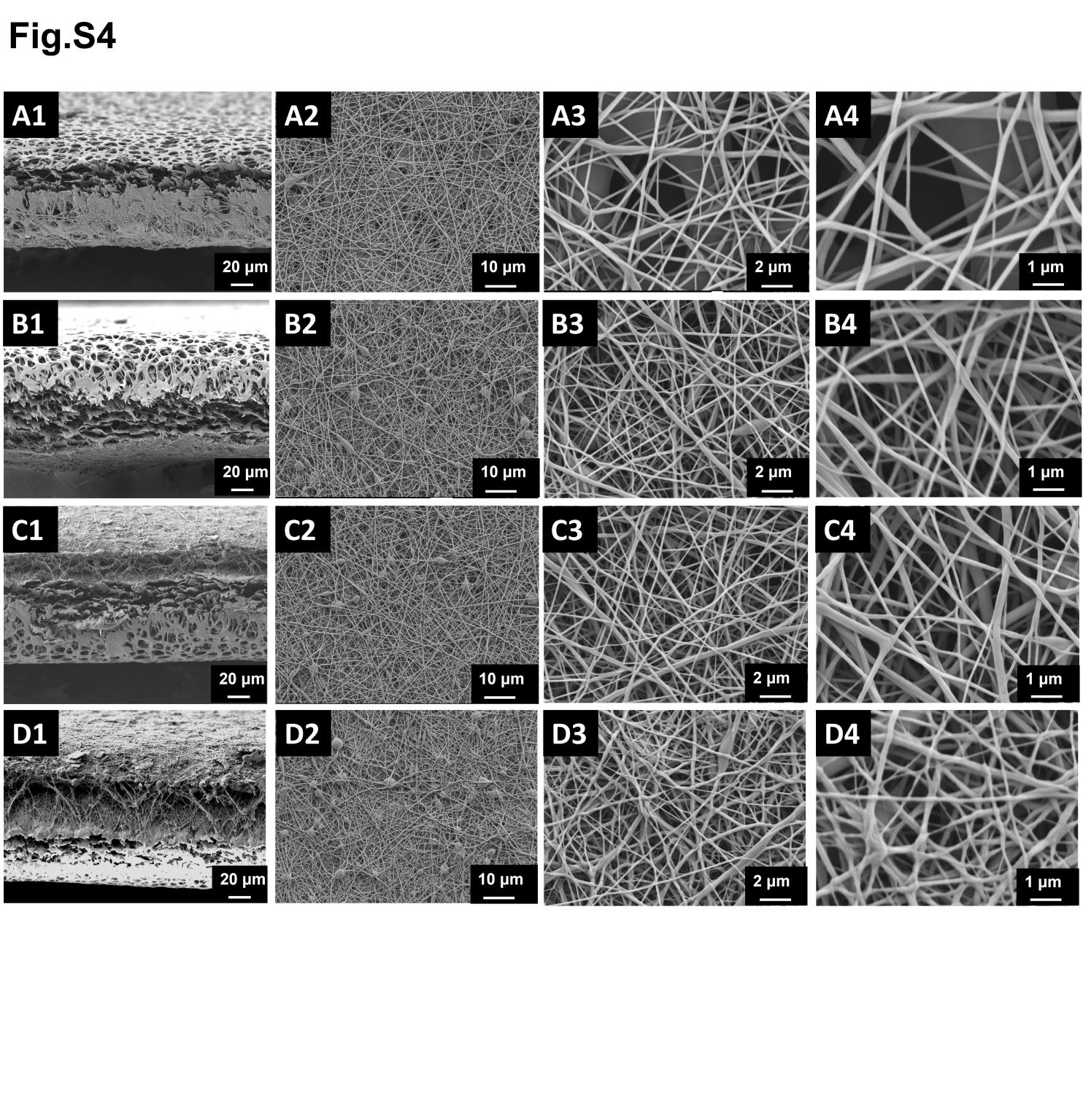


**Figure S4| Morphology characterization of the proposed liquid diode with different thickness.** SEM images showing cross-sectional morphology of liquid diode with (A1) 1 μm, (B1) 10 μm, (C1) 30 μm and (D1) 60 μm thickness TPU membrane at 500 times magnification. SEM images displaying the surface morphology of the liquid diode with TPU membranes of thicknesses (A) 1 μm, (B) 10 μm, (C) 30 μm, and (D) 60 μm at (B2-D2) 1,000 times magnification, (B3-D3) 5,000 times magnification, and (B4-D4) 10,000 times magnification.


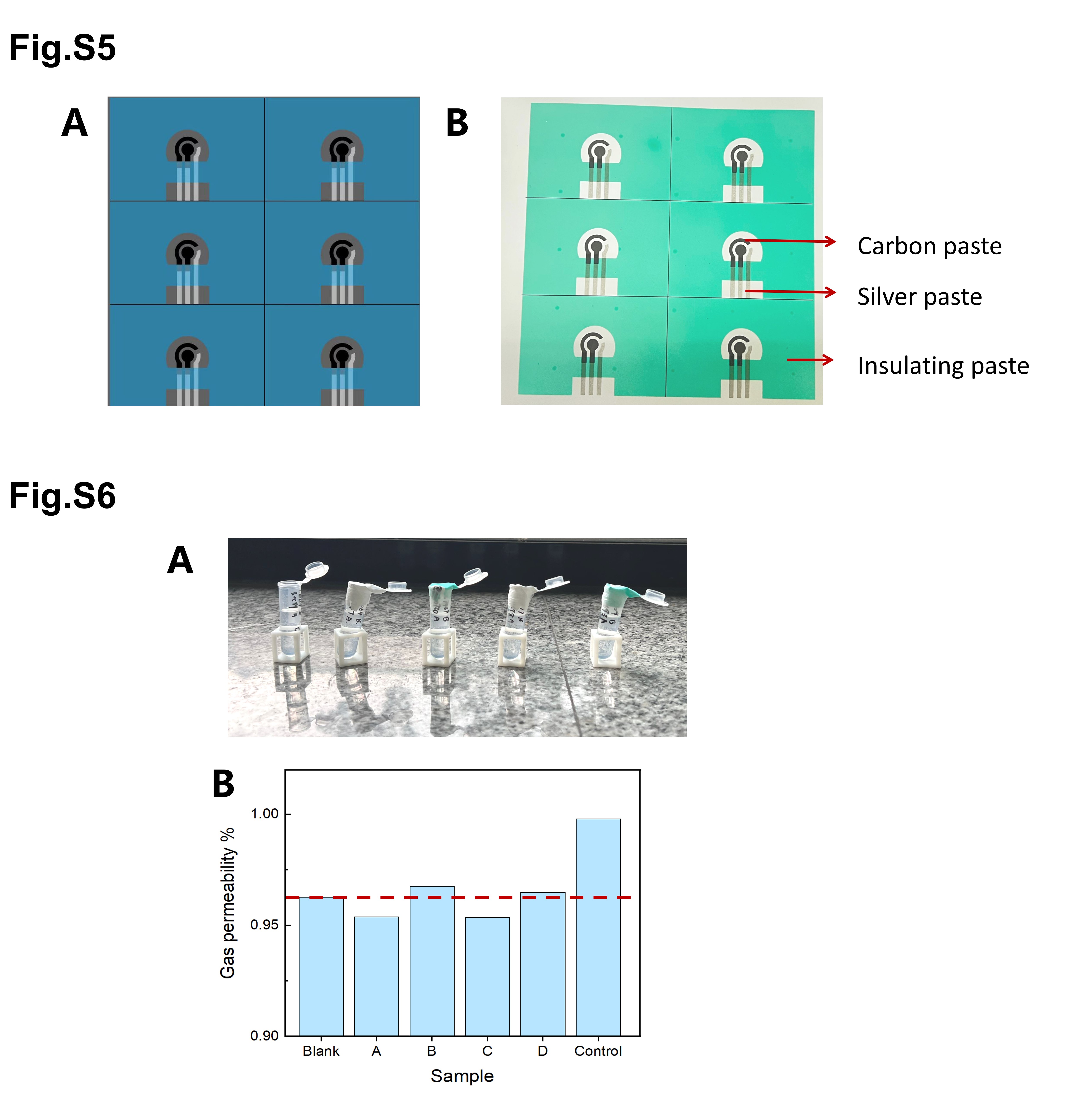


**Figure S5| Screen printed carbon electrodes (SPCE) on the liquid diode.** (A) Design of SPCE on the liquid diode. (B) Photo of SPCE on the liquid diode. The working electrodes and counter electrodes were made of carbon paste, while the reference electrodes and electrical leads were composed of silver paste. The remaining areas were covered by insulating paste.


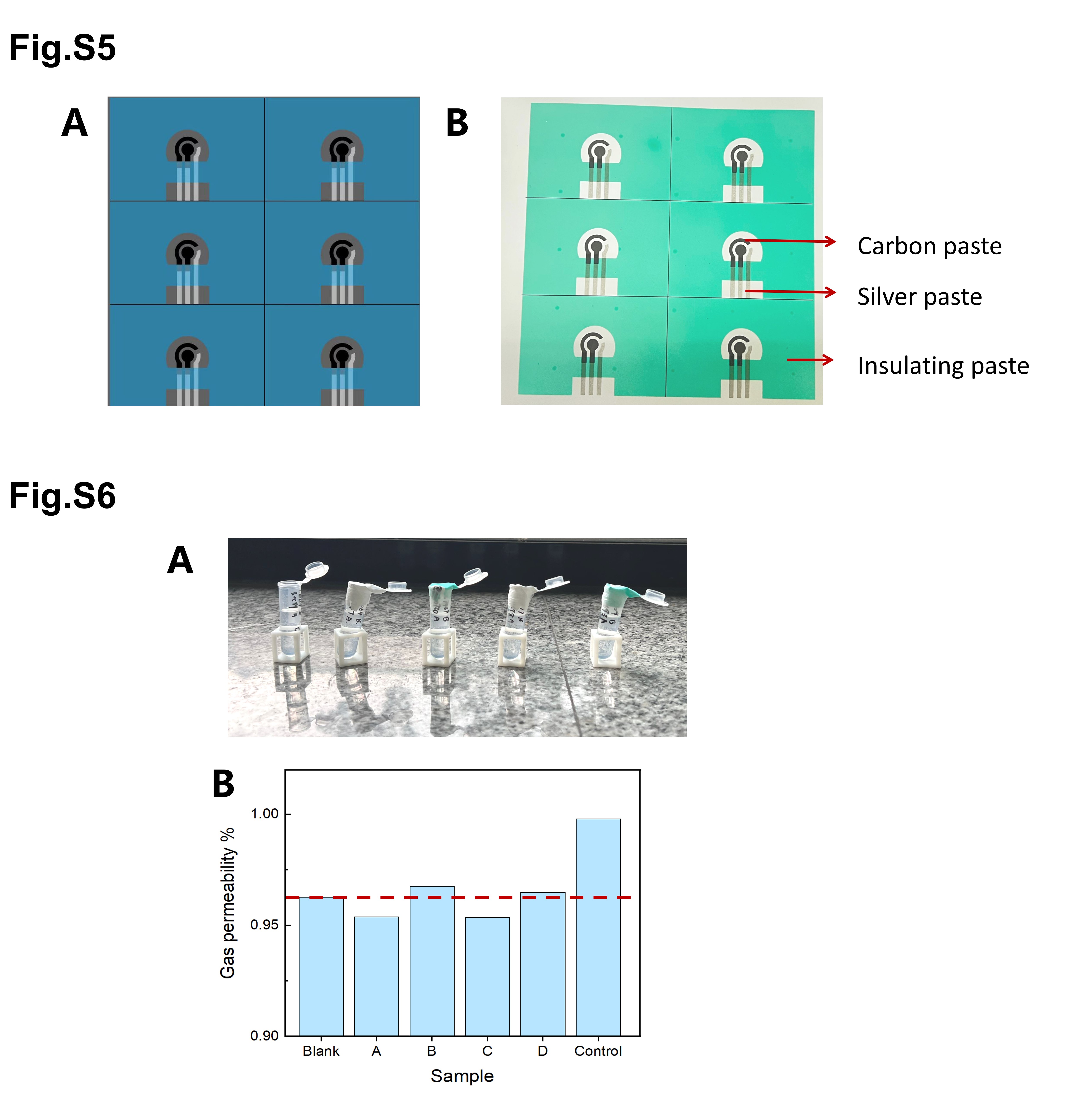


**Figure S6| Gas permeability of liquid diodes integrated with SPCE.** (A) Photo of the gas permeability test of liquid diodes integrated with SPCE. The container of the blank group was fully open, while the container of the sealed group was completely sealed with non-breathable plastic caps. The test groups were covered by bare PES membrane, PES membrane integrated with SPCE, bare liquid diode, and liquid diode integrated with SPCE, respectively. (B) Histogram displaying the calculated gas permeability of each group. A, B, C, D represents the permeability of bare PES membrane, PES membrane integrated with SPCE, bare liquid diode, and liquid diode integrated with SPCE, respectively.


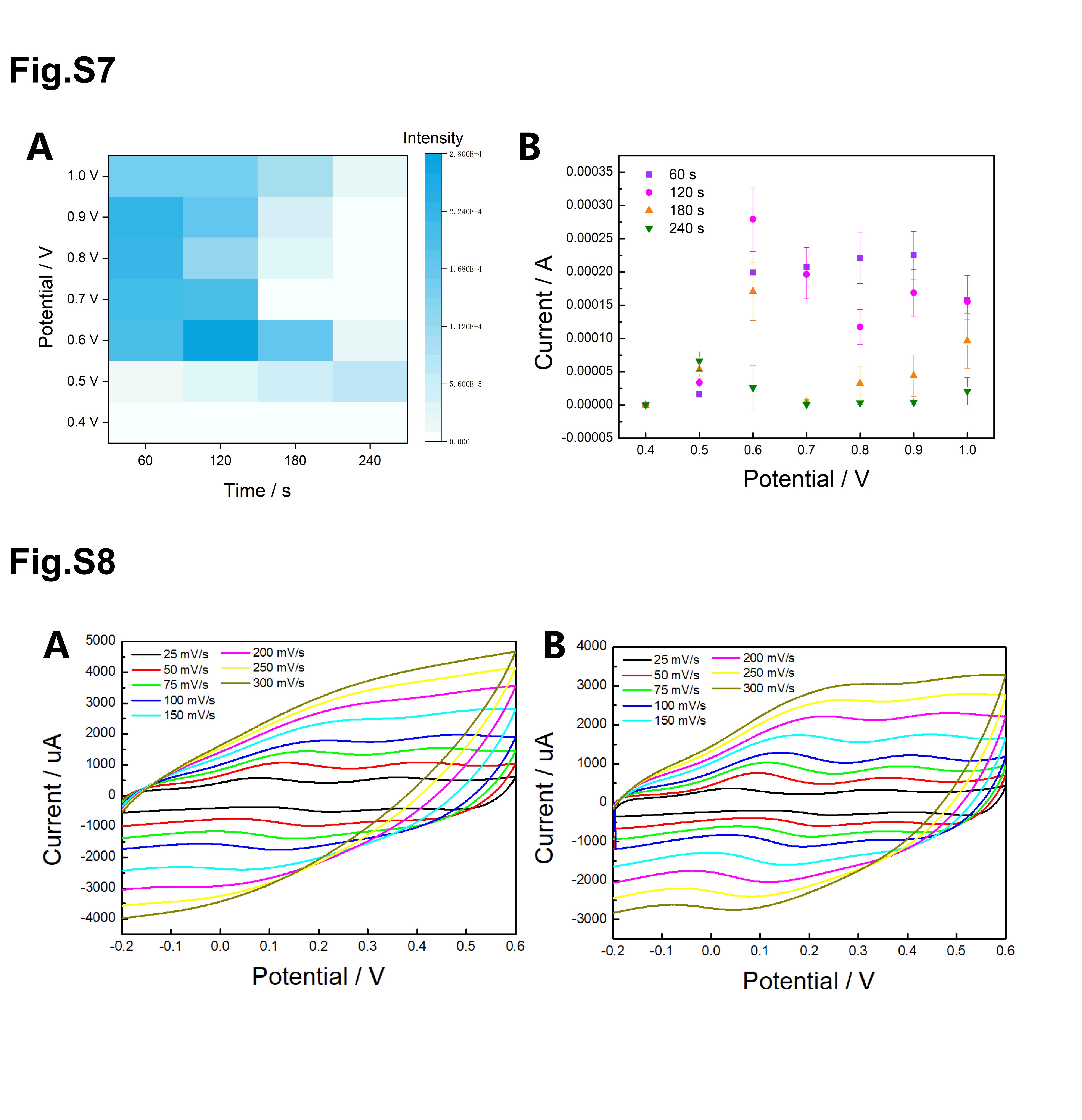


**Figure S7| Condition optimization of electrodeposition potentials and time for M-PANI fabrication.** (A) Heat graph of the peak oxidation current of cyclic voltammetry under different electrodeposition potentials ranging from 0.4 V to 1.0 V with intervals of 0.1 V, for various deposition times ranging from 60 s to 240 s. (B) Histogram displaying the peak oxidation current of cyclic voltammetry under different electrodeposition potentials ranging from 0.4 V to 1.0 V with intervals of 0.1 V, for various deposition times ranging from 60 s to 240 s.


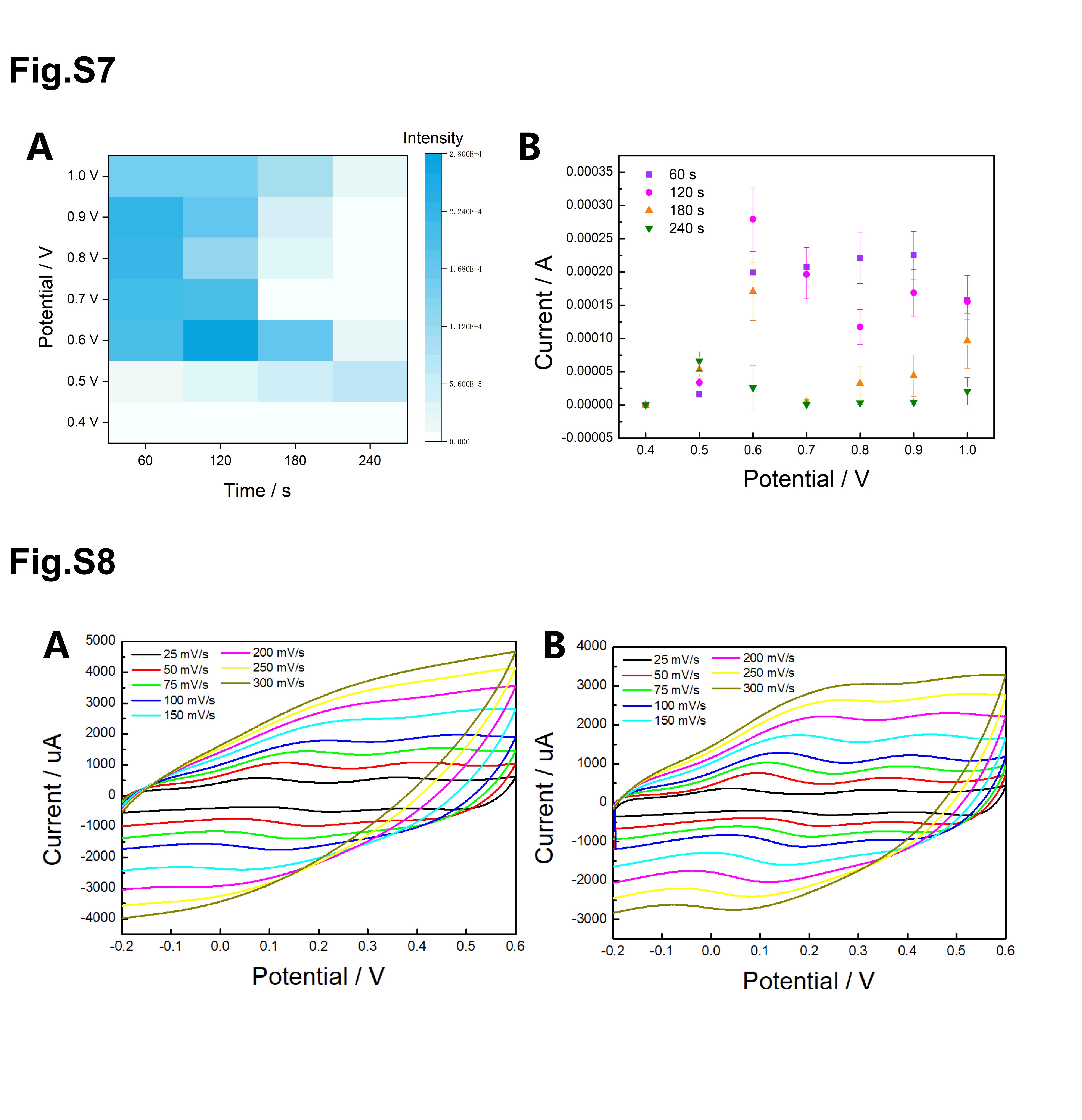


**Figure S8| Reaction kinetic analysis of L-PANI/SPCE and M-PANI/SPCE.** Cyclic voltammetry curves of (A) L-PANI/SPCE and (B) M-PANI/SPCE under increased scanning rates ranging from -0.2 V to 0.6 V. The scanning rate increased from 25 mV/s to 100 mV/s with a 25 mV/s interval, and from 100 mV/s to 300 mV/s with a 50 mV/s interval.


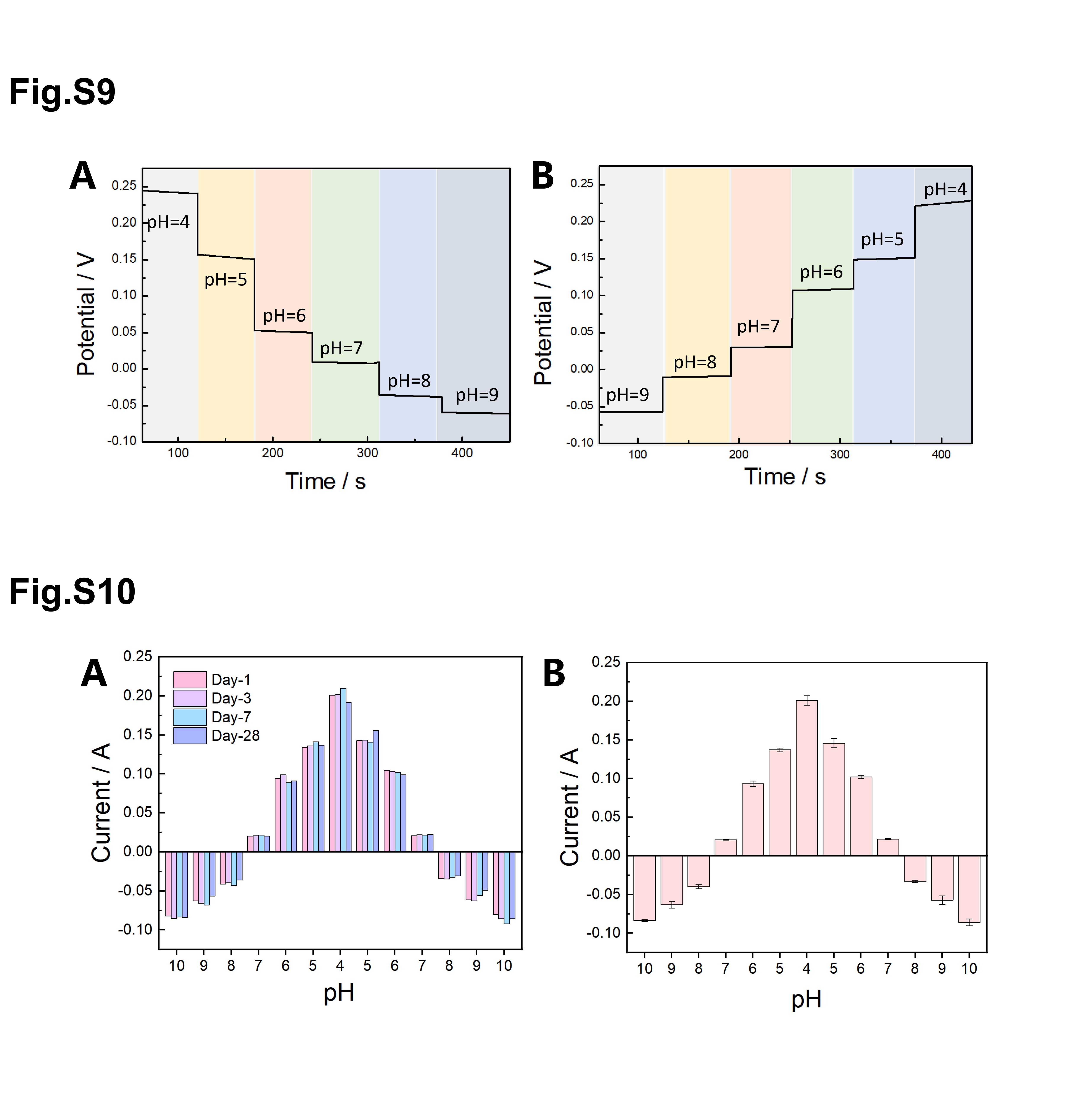


**Figure S9| Dynamic response of M-PANI/SPCE in various pH solutions**. (A) Open circuit potential-time (OCPT) curves of M-PANI/SPCE under different pH solutions, ranging from pH 4.0 to pH 9.0. (B) OCPT curves of M-PANI/SPCE under different pH solutions, ranging from pH 9.0 to pH 4.0.


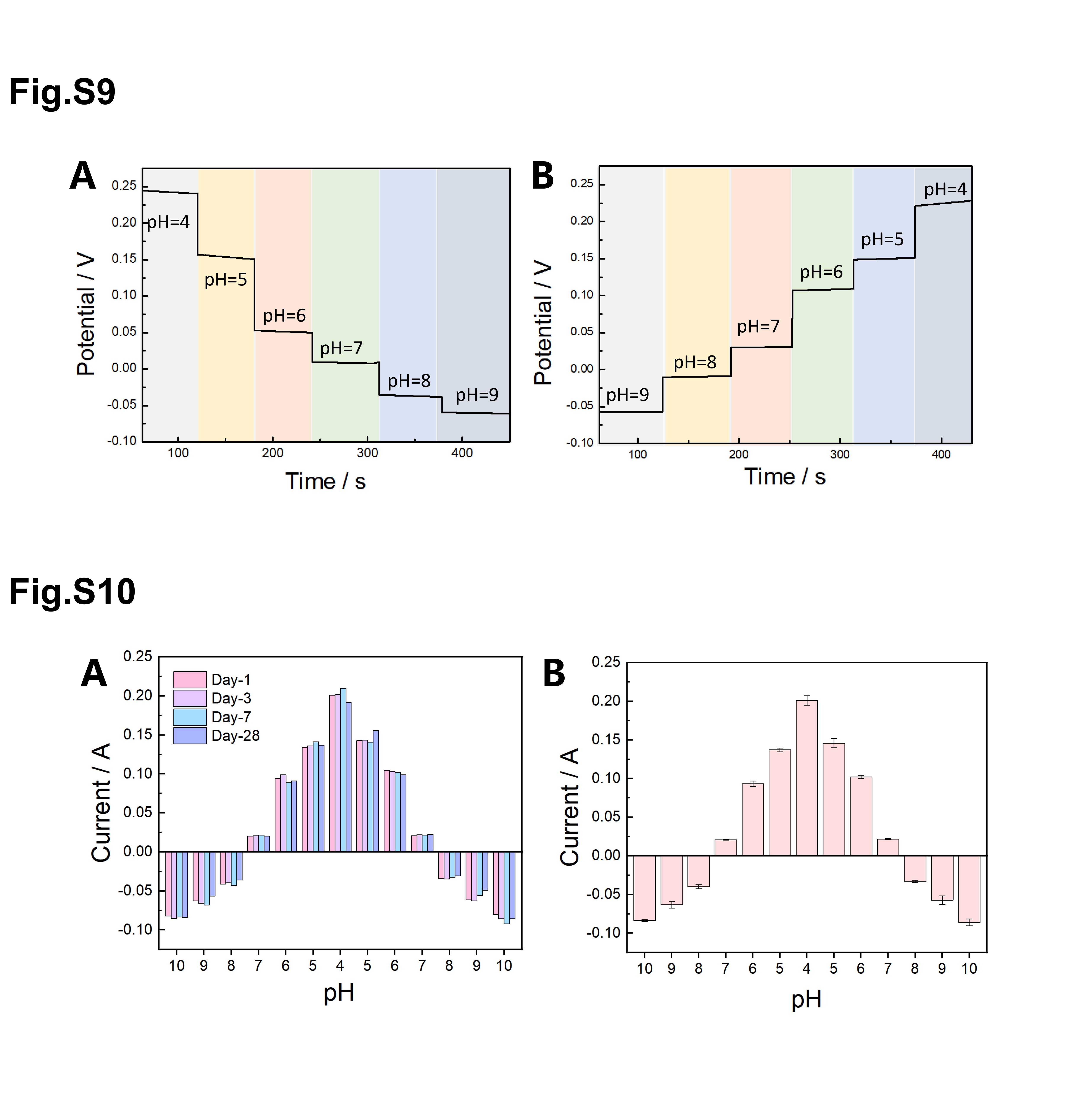


**Figure S10| Long-term stability of M-PANI/SPCE for storage.** (A) Histogram of OCPT response of M-PANI/SPCE under different pH solutions ranging from pH 10.0 to pH 4.0 in a continuous loop at the 1^st^, 3^rd^, 7^th^, and 28^th^ day. (B) Histogram of calculated OCPT response of M-PANI/SPCE under different pH solutions varying from pH 10.0 to pH 4.0 in a continuous loop for 28 days of storage.


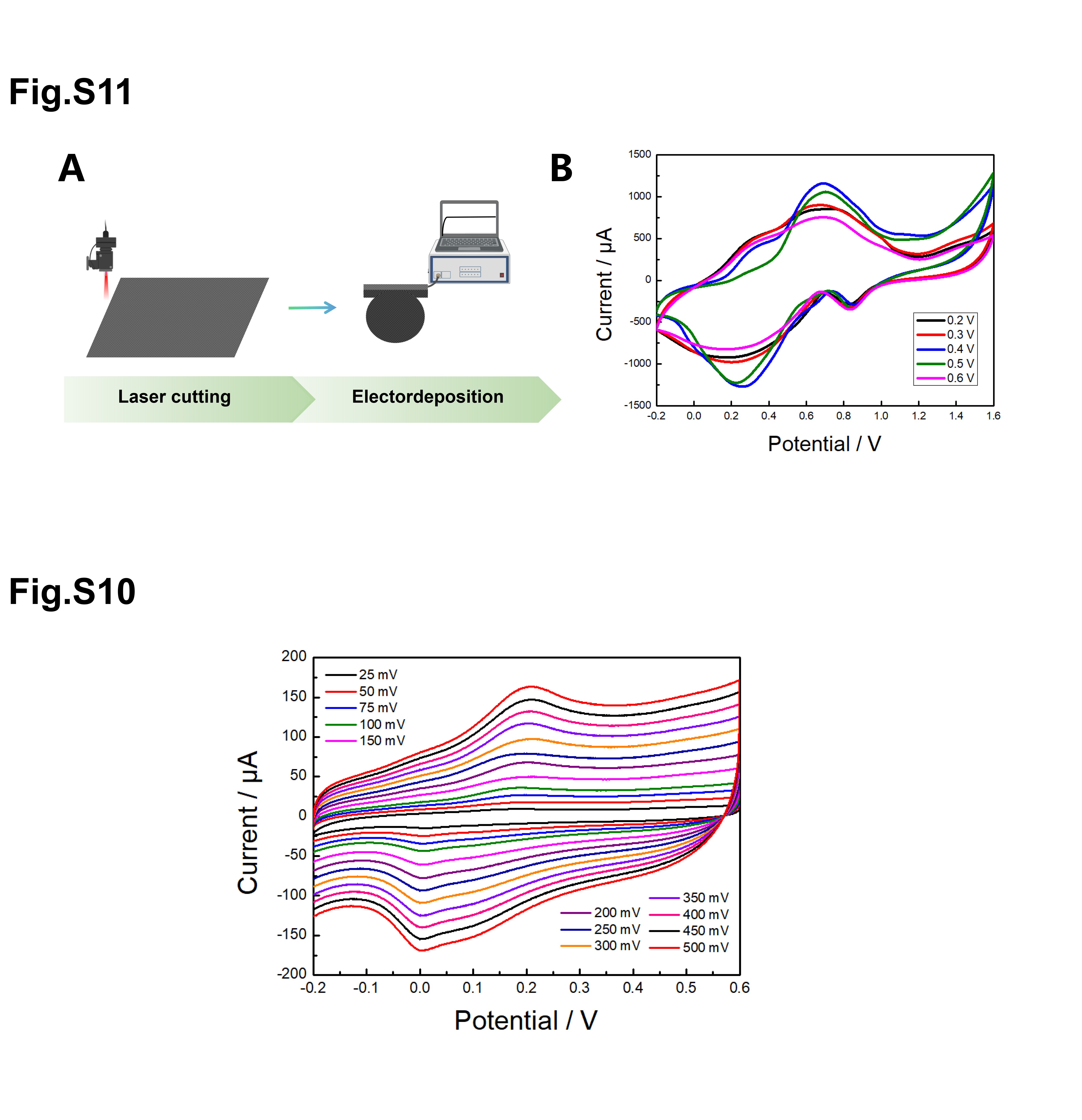


**Figure S11|** **Transfer of M-PANI based sensing interface on carbon fiber paper (CFP).** (A) Schematic diagram of the transfer of M-PANI based sensing interface on CFP including laser cutting and electrodeposition. (B) Cyclic voltammetry curve of M-PANI/CFP fabricated under various potentials ranging from 0.2 V to 0.6 V.


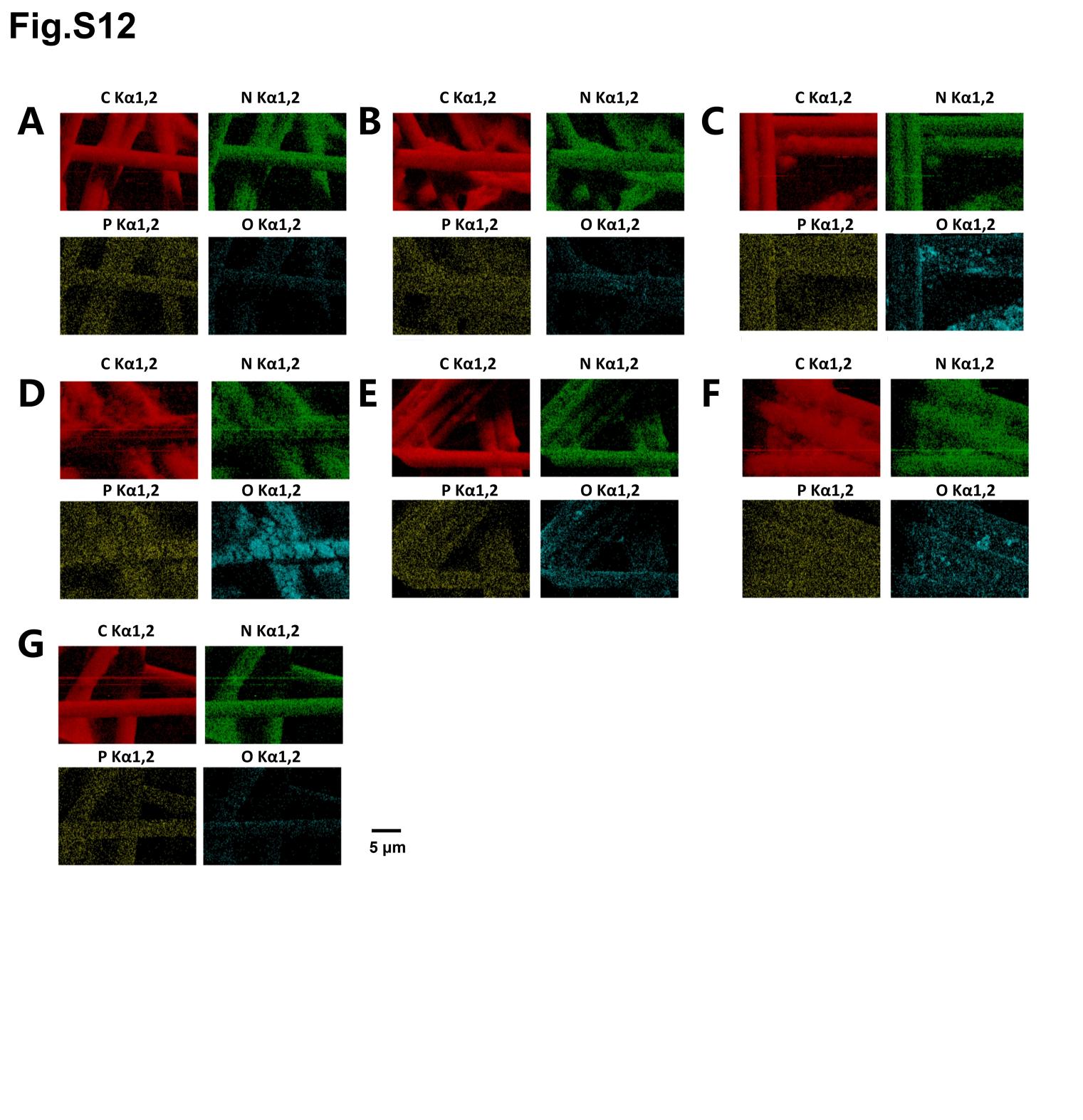


**Figure S12| Element distribution of** **M-PANI/CFP fabricated at different potentials.** EDS images of M-PANI/CFP fabricated under (A) 0.1 V, (B) 0.2 V, (C) 0.3 V, (D) 0.4 V, (E) 0.5 V, (F) 0.6 V, and (G) 0.7 V for 300 s, showing the distribution of carbon, nitrogen, phosphorus and oxygen.


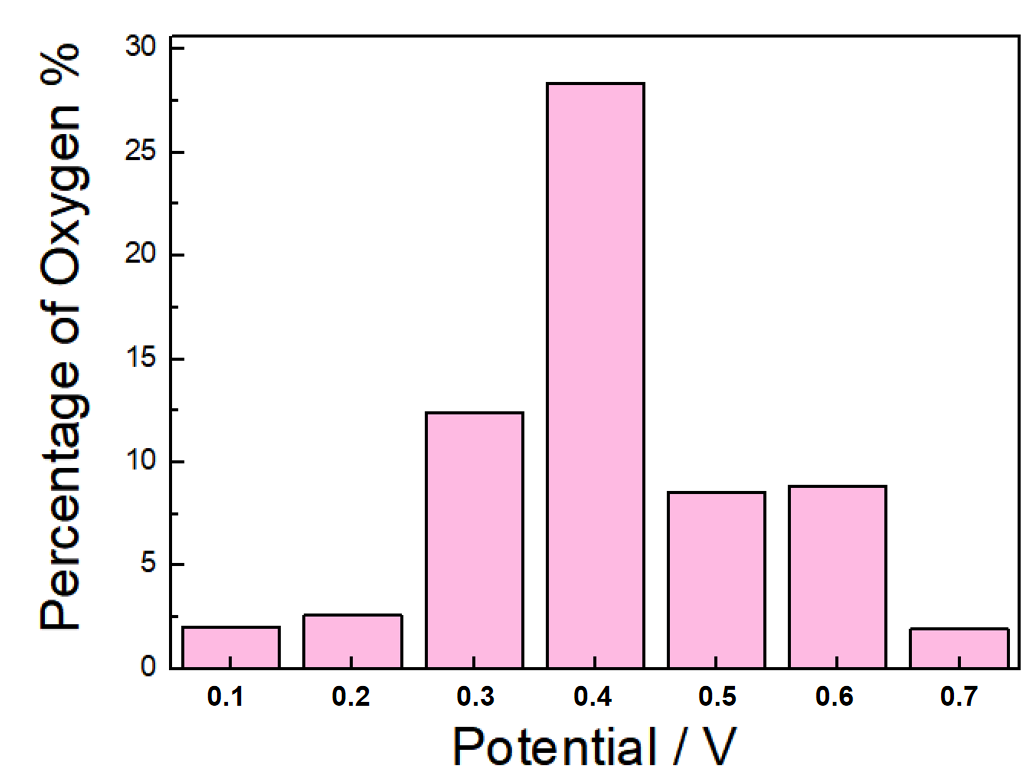


**Figure S13| Oxygen percentage of M-PANI/CFP fabricated at different potentials.** Histogram of oxygen percentage of M-PANI/CFP fabricated at different potentials ranging from 0.1 V to 0.7 V.


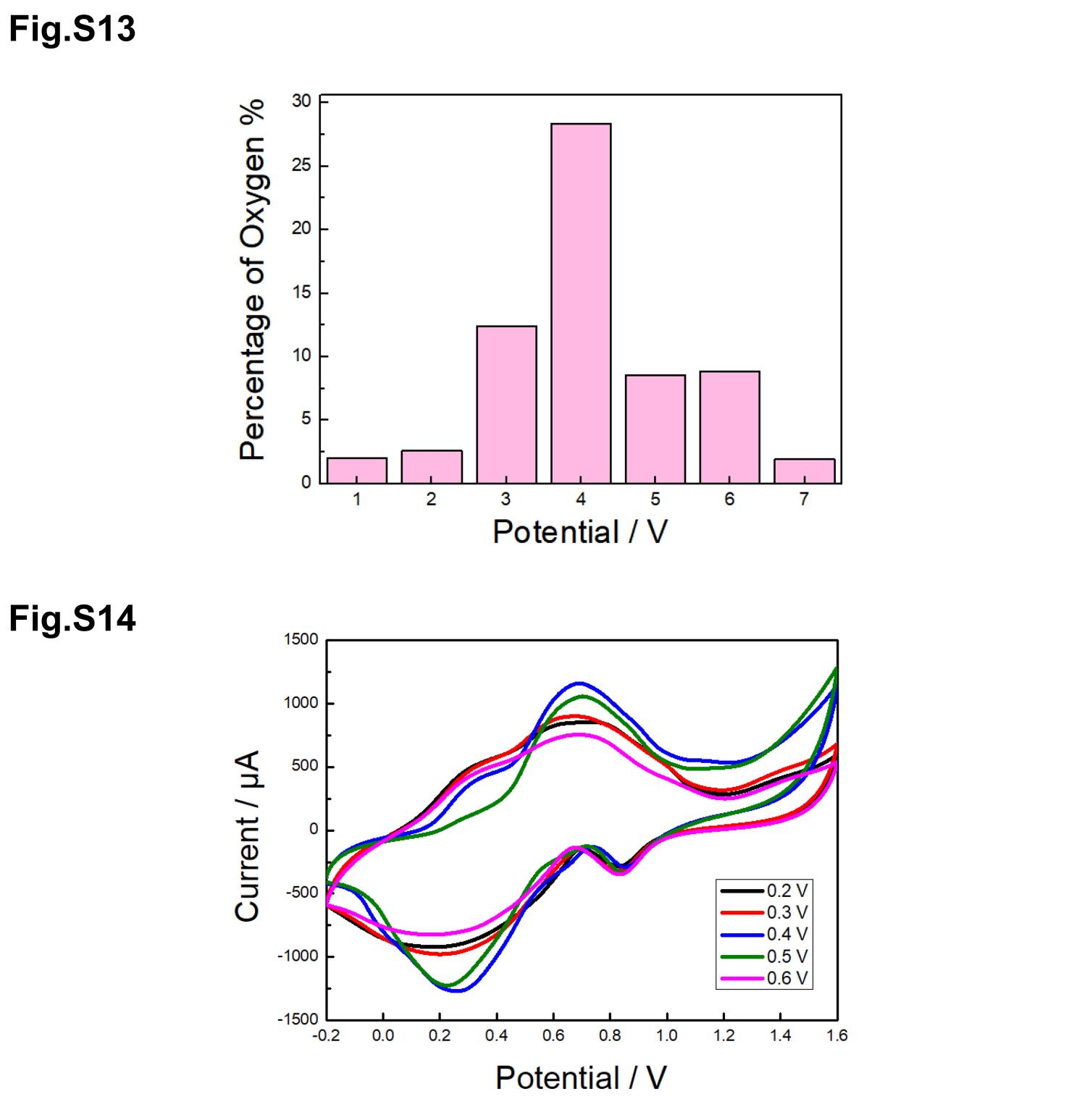


**Figure S14| Cyclic voltammetry tests of M-PANI/CFP fabricated at various potentials.** Cyclic voltammetry curve of M-PANI/CFP fabricated under various potentials ranging from 0.2 V to 0.6 V.


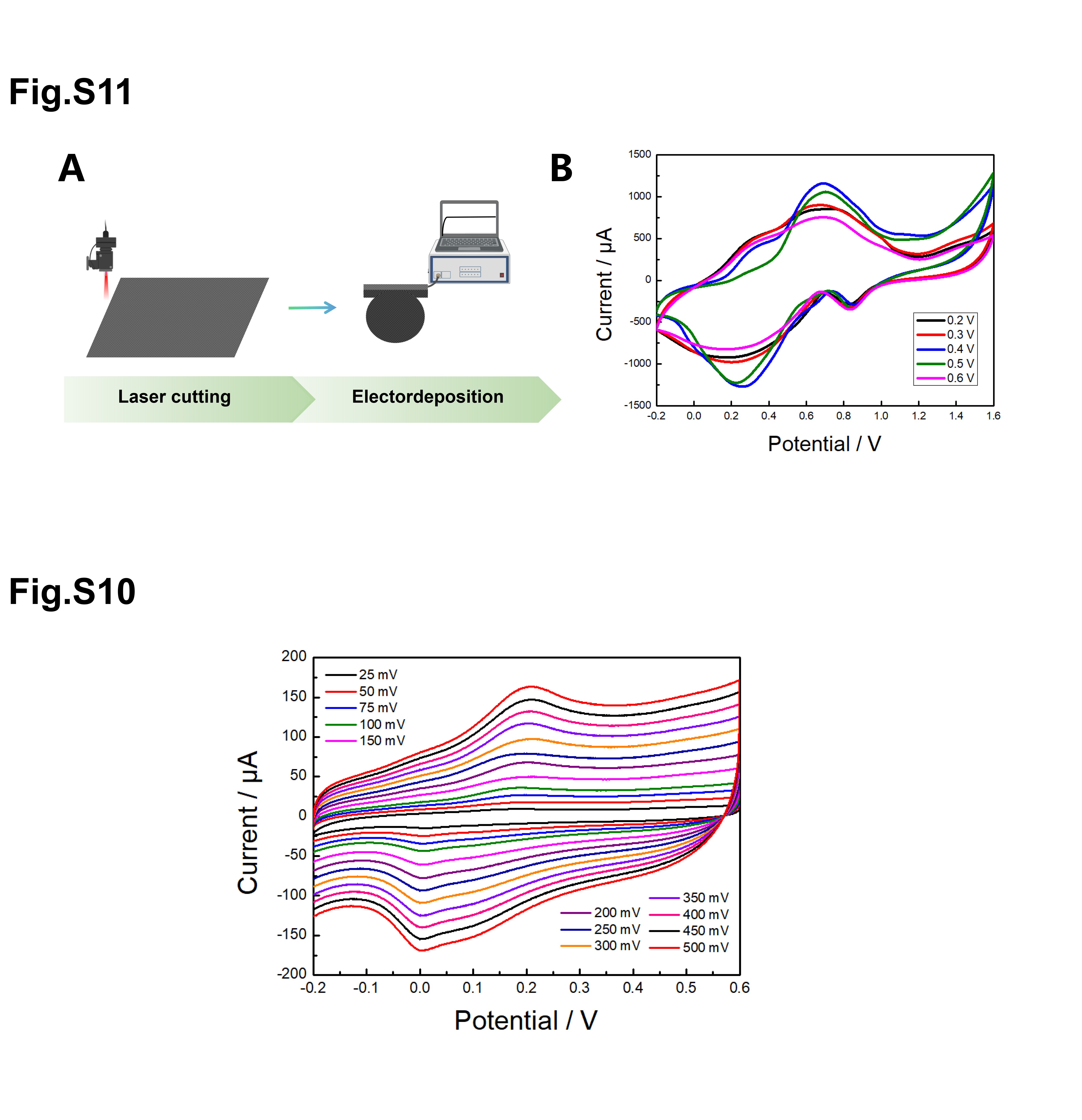


**Figure S15| Reaction kinetic analysis of M-PANI/CFP.** Cyclic voltammetry curves of M-PANI/CFP under increased scanning rates ranging from -0.2 V to 0.6 V. The scanning rate increased from 25 mV/s to 100 mV/s with a 25 mV/s interval, and from 100 mV/s to 500 mV/s with a 50 mV/s interval.

**Reference**

1. Campos M and Braz Bello Jr. Mechanism of conduction in doped polyaniline. *J. Phys. D: Appl. Phys*. **30**, 1531 (1997).
2. Yang D, Wang J, Cao Y, et al. Polyaniline-based biological and chemical sensors: Sensing mechanism, configuration design, and perspective. *ACS Appl. Electron. Mater.* **5(**2): 593-611 (2023).
